# Taxonomic and functional diversity of benthic macrofauna associated with rhodolith beds in SE Brazil

**DOI:** 10.7717/peerj.11903

**Published:** 2021-07-29

**Authors:** Patricia Sarcinelli Stelzer, Ana Carolina A. Mazzuco, Luiz Eduardo Gomes, João Martins, Sergio Netto, Angelo F. Bernardino

**Affiliations:** 1 Department of Oceanography, Universidade Federal do Espírito Santo, Vitoria, Espirito Santo, Brazil; 2 Laboratório de Ciências Marinhas, Universidade do Sul de Santa Catarina, Tubarão, Santa Catarina, Brazil

**Keywords:** Rhodolith, Benthos, Ecology, South Atlantic, Macrofauna

## Abstract

Rhodoliths are free-living and morphologically diverse marine calcareous algae commonly distributed over the continental shelf seafloor. They increase the seabed structural complexity and are of potential value as feeding and reproductive grounds for a myriad of marine fauna. The higher structural seabed complexity within rhodolith beds may also increase benthic diversity by creating microhabitats, but this relationship has been rarely explored within rhodolith beds worldwide. Here we compared benthic macrofaunal (>500 µm) structure on rhodolith beds (nodule epifauna) and within unconsolidated sediments (sediment infauna) under high and low-density beds to test whether rhodolith bed density and nodule morphology influenced macrofaunal assemblages. We observed that macrofaunal density on nodules (2538 ± 288.7 ind·m^−2^) was 15-fold higher when compared to sediments under those beds (166 ± 38.8 ind·m^−2^). Rhodolith bed density was positively related to macrofaunal density, composition, and functional diversity on the rhodoliths. Low-density beds (61 ± 27.1 nodules·m^−2^) with discoid-shape nodules were dominated by peracarid crustaceans whereas high-density beds (204 ± 18.7 nodules·m^−2^) with spheroidal nodules were dominated by Annelid polychaetes. The sediment macrofauna was also positively influenced by the density of rhodolith nodules, which increased sediment carbonate and organic quality (protein and lipids) under high-density beds. Macrofaunal functional diversity was generally higher on rhodoliths, with low similarity (low nestedness) and high taxa turnover between macrofaunal assemblages of rhodoliths and sediments. These findings indicate that rhodolith beds provide an unique habitat for benthic macrofaunal communities, with exclusive functional and taxonomic richness that are likely not typical in the unconsolidated sediment below these beds in SE Brazil. This study highlights the importance of protecting rhodolith beds from multiple sources of anthropogenic disturbance and exploration on continental shelves.

## Introduction

Rhodoliths are nodules primarily composed of non-geniculate free-living red algae that are distributed globally over continental shelves and oceanic islands (Foster, 2001; Amado-Fiho et al., 2017). They occur in areas with moderate hydrodynamics that prevents burial caused by particle sedimentation and protects them from physical impact, fragmentation, and removal by strong currents (Hinojosa-Arango, Maggs & Johnson, 2009; McConnico et al., 2017). The structures formed by the accumulation of these nodules are known as rhodolith beds, which typically occur in waters shallower than 150 m with favorable temperature and irradiance for photosynthetic, respiratory, and calcification processes (Riosmena-Rodríguez, 2017). These beds create a three dimensional structure over the seafloor, modifying its physical characteristics and creating new microhabitats for many marine species (Steller et al., 2003; Berlandi, de O. Figueiredo & Paiva, 2012; Teichert, 2014; Qui-Minet et al., 2018). Besides hosting a diverse range of benthic organisms, rhodolith beds also provide a number of ecosystem services. They serve as refuge and nursery grounds for marine species, some of them commercially important like scallops, crabs, and fish (Kamenos, Moore & Hall-Spencer, 2004; Steller & Cáceres-Martínez, 2009; Riosmena-Rodriguez & Medina-López, 2010; Costa et al., 2020). Rhodolith beds are likely one of the most important benthic habitats on Brazil’s continental shelf in terms of biodiversity and heterogeneity, harboring rare and endemic species of macroalgae, polychaetes, and ictiofauna (Gherardi, 2004; Amado-Fiho et al., 2017). Therefore, these living beds contribute significantly to the increase of regional species richness and diversity (Steller et al., 2003; Teichert, 2014), suggesting that they are of critical value for biodiversity conservation (Biomaerl team, 2003; Crain & Bertness, 2006).

Anthropogenic pressures on rhodolith beds (*i.e*. fishing, climate change, mining interests, and offshore oil and gas operations) are expected to increase over the coming decades, threatening the long-term survival of these ecologically-important habitat (Hall-Spencer & Moore, 2000; McCoy & Kamenos, 2015; Horta et al., 2016; Almada & Bernardino, 2017; Schubert et al., 2019; Simon-Nutbrown et al., 2020; Sissini et al., 2020). Understanding the spatial drivers that influence benthic biodiversity in rhodolith beds is thus critical for conservation planning over areas with multiple industrial interests (Bernardino & Sumida, 2017). In addition to understanding benthic taxonomic diversity associated with rhodoliths, determining the species traits and their spatial variability can help to quantify the benthic functional diversity (Mokany, Ash & Roxburgh, 2008), providing information on the dynamics and uniqueness of communities or ecosystems (Violle et al., 2007; Mouchet et al., 2010). In this context, functional indices that represents species distribution and its functionalities complement diversity and taxonomic indexes to differentiate the structure and function of ecological communities within the functional trait space (Petchey & Gaston, 2006). Rhodolith beds are habitats already known to host a high taxonomic diversity and are a priority for conservation on continental margins (Hall-Spencer, 1998; Grall & Hall-Spencer, 2003; Nelson, 2009), but their functional diversity has not been investigated in detail on Brazilian beds.

The benthic macrofauna has a crucial role in maintaining important ecosystem services in the ocean, including energy-mass exchange and nutrient cycling between the water column and sediments (Snelglove & Buttman, 1995). The interaction between benthic fauna and the seafloor plays a key role in determining the composition and diversity of benthic assemblages (Snelglove & Buttman, 1995). As a result, habitat complexity is considered the main driver of benthic community structure and ecological functions. The increase of seafloor complexity within rhodolith beds are then expected to result on a higher diversity and abundance of benthic species (Buhl-Mortensen et al., 2012; Kovalenko, Thomaz & Warfe, 2012; Yanovski, Nelson & Abelson, 2017) when compared to sand bottoms of lower complexity. These effects have been observed in a number of rhodolith beds globally, supporting that rhodoliths play an important role to overall biodiversity over continental margins (Hily, Potin & Floc’h, 1992; Steller et al., 2003; Veras et al., 2020). The structural heterogeneity of rhodolith beds may also vary spatially and temporally from both natural and anthropogenic factors (Hall-Spencer & Moore, 2000; Steller et al., 2003; Fredericq et al., 2014).

In Brazil, the fauna associated with rhodolith beds indicates a high diversity of species across extensive areas of the continental shelf (Villas-Boas et al., 2009; Horta et al., 2016; Amado-Fiho et al., 2017; Carvalho et al., 2020). Although the biodiversity associated with Brazilian rhodolith beds has been previously assessed, this study investigates the drivers of both rhodolith and sediments macrofaunal diversity, composition and functional dynamics thus providing a new perspective of the effects of rhodoliths over sedimentary macroinfaunal assemblages. Here we evaluated how the structural change in rhodolith nodules are associated to macrobenthic assemblages at high- and low-density rhodolith beds in SE Brazil. Considering the important role of rhodoliths as ecosystem engineers, this study examines how macrofauna assemblages change across habitats and beds with different nodule densities and morphology, addressing two hypotheses: (I) Macrofaunal assemblages will have a higher diversity associated with rhodolith nodules when compared to the unconsolidated sediment under them, and (II) the density of rhodolith beds will be important to spatial patterns of benthic diversity in nodules and in the underlying sediments.

## Materials & methods

### Study area and sampling design

**Figure 1:**
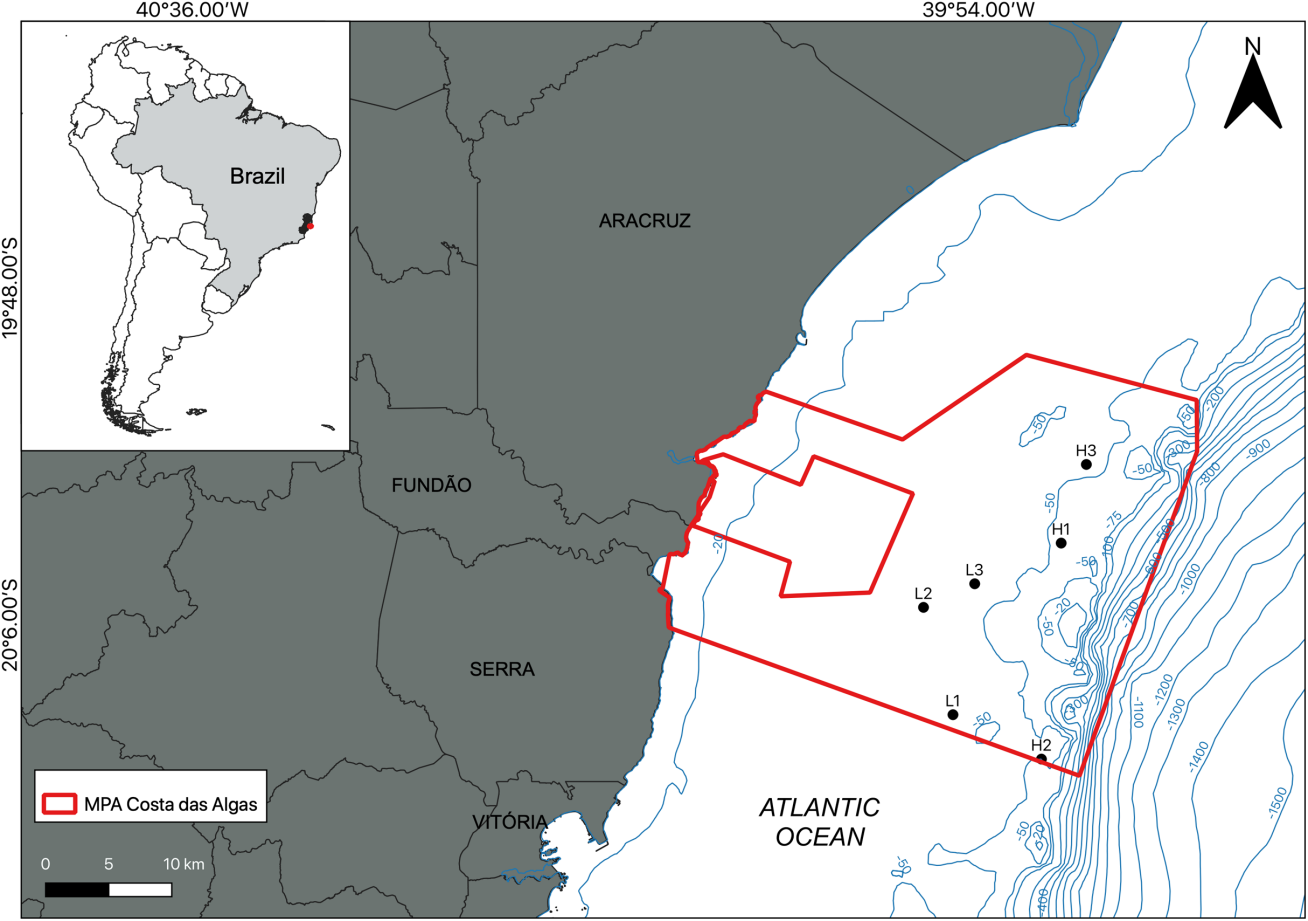
Location of the study area. Map of the Marine Protected Area (MPA) Costa das Algas (larger polygon) and the six sampled stations on the SE continental shelf of Brazil. Bathymetric isobaths are shown in blue.

The study area is located within the limits of the Costa das Algas Marine Protected Area (MPA) on the Eastern Marine Ecoregion of Brazil (Fig. 1; Table 1; Spalding et al., 2007). This is a tropical region characterized by rainy summers, with predominantly NE and E winds, and dry winters (Bernardino et al., 2015). The continental shelf on Eastern Brazil is influenced by the Tropical Water (TW) of the Brazil Current, with temperatures above 22 °C and salinities above 36 (Mazzuco et al., 2019), and eventual seasonal summer upwelling of the South Atlantic Central Water (SACW) into the shelf, with temperatures between 6 °C and below 20 °C and salinities between 34.6 and 36 (Quintana et al., 2015; Mazzuco et al., 2019). The continental shelf on the Espírito Santo basin includes a mixed system of terrestrial and carbonate sediments with rhodolith beds extending down to the shelf break at depths over 80 m (Figueiredo et al., 2015; Holz et al., 2020).

**Table 1:** Sampling stations and environmental data.

Station	Z (m)	D_SC_ (m)	B_T_ (°C)	B_S_	ND (N·m^−2^)	V_I_ (cm^3^·m^−2^)	D_M_ (cm)	Ramification				TOM (%)	Gravel (%)	Sand(%)	Silt(%)	Carbonate (%)
(Lat/Long)								I	II	III	IV					
H1(20°01′36.5″S/39°49′35.1″W)	54.0	34	22.2	38	171	153.3(±69.4)	2.8(±0.0)	0.05(±0.03)	0.11(±0.08)	0.18(±0.005)	0.66(±0.05)	2.9(±0.3)	28.4(±9.1)	70.1(±8.9)	1.5(±0.3)	5.4(±0.3)
H2(20°13′8.4″S/39°50′38.4″W)	54.8	30	20.8	37.9	206	346.6(±34.2)	3.9(±0.1)	0.04(±0.07)	0	0	0.99(±0.01)	3.4(±0.1)	19.5(±6.3)	78.1(±6.5)	2.4(±0.4)	5.0(±0.1)
H3(19°57′24.6″S/39°48′14.4″W)	50.6	33	20.2	37.7	236	436.6(±103.1)	3.7(±0.1)	0.03(±0.03)	0.02(±0.02)	0	0.94(±0.03)	4.7(±0.7)	19.8(±4.9)	78.6(±4.8)	1.6(±0.2)	7.2(±0.3)
L1(20°10′46.2″S/39°55′21.96″W)	47.2	20	21.7	38	104	246.6(±30.0)	3.8(±0.3)	0.01(±0.02)	0.01(±0.02)	0	0.98(±0.03)	2.9(±0.3)	7.0(±1.1)	91.4(±1.2)	1.6(±0.2)	4.0(±0.3)
L2(20°05′02.4″S/39°56’56.4’’W)	39.5	25	20.9	37.8	68	368.3 (±132.9)	5.3(±0.2)	0.02(±0.03)	0.02(±0.03)	0.16(±0.06)	0.80(±0.09)	4.3(±0.3)	18.6(±1.0)	79.6(±0.7)	1.8(±0.3)	5.3(±0.2)
L3(20°03’46.8’’S/39°54’12.3’’W)	45.6	–	19.1	37.3	11	4.0(±9.0)	3.8(±0.2)	0	0.01(±0.01)	0.01(±0.02)	0.98(±0.02)	–	–	–	–	–

**Note:**

Sampling station location, maximum depth (Z), Secchi’s depth (D_SC_), bottom temperature (B_T_) and salinity (B_S_), number of nodules (ND), mean internal volume (V_I_), mean diameter (D_M_), and pattern of ramification (%, type I, II, III, and IV) of the rhodolith nodules. Mean sedimentary characteristics (% contribution) of total organic matter (TOM), gravel, sand, silt, and carbonate. Standard error shown within parentheses.

**Figure 2:**
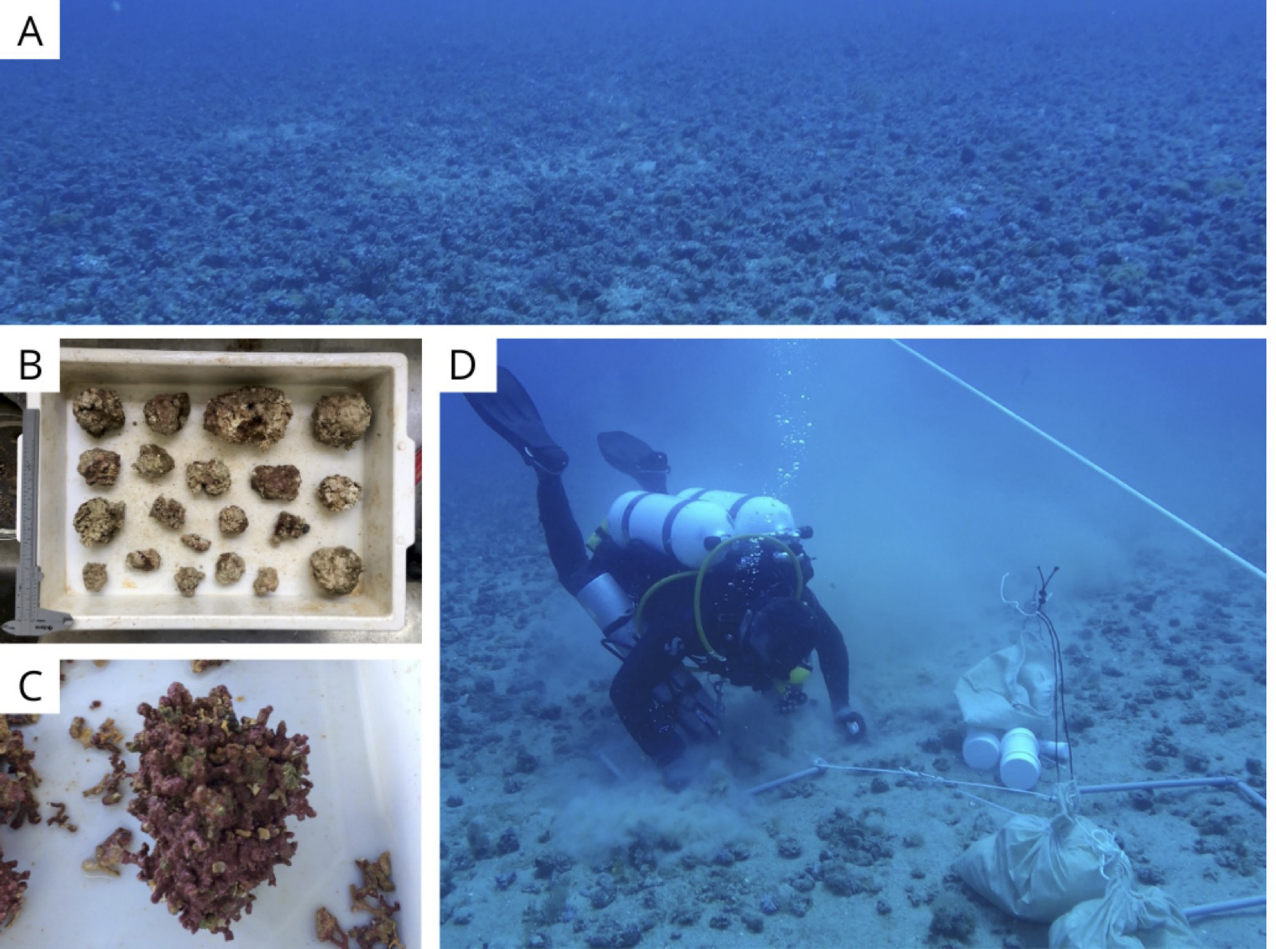
Sampling sites and methods. (A) High-density rhodolith beds within the study area, (B–C) a range of rhodoliths sampled in this study, and (D) SCUBA sampling from this study.

Sampling was carried out by SCUBA diving in January 2019 and the density of the rhodolith beds was a determining factor of the sampling design. Based on preliminary images of the area, sampling stations were classified into two categories (beds): high-density (H1, H2, and H3) and low-density (L1, L2, and L3; Fig. 1). High density beds had 100% of the seafloor covered by rhodoliths, and low-density beds had scattered patches of sediment between rhodolith nodules. The differences in bed structure were further confirmed by measurements of rhodolith morphology, internal volume, and ramification (branch density, see below). Abiotic metadata (temperature, salinity, depth, and water visibility) were obtained at the time of sampling using a CTD and Secchi disk. In each station, scuba divers sampled manually all rhodoliths on the surface (50–100 rhodoliths in high-density beds; 4–40 rhodoliths in low-density beds) within a 0.25 m^2^ quadrat from three replicates randomly distributed along a 20 m random transect (Fig. 2). Occasional megafaunal organisms were observed (macroalgae, ophiuroids) but were not sampled and were thus excluded from our analysis. Triplicate samples of the underlying unconsolidated sediment within each quadrat were collected using PVC corers of 10 cm in diameter (10 cm depth) with sealing lids. The rhodolith nodules were packed in cloth bags (<0.5 mm mesh) and sealed to prevent loss of macrofauna during recovery on board, where they were immediately fixed with formaldehyde (10%) and borax to avoid carbonate degradation. Macrofaunal invertebrates in the sediments were also fixed with formaldehyde (10%) and borax. In addition to the biological data, sediment underlying the rhodolith beds was also sampled for grain size, organic matter, and biopolymers analysis using triplicate corers (10 cm diameter), preserved in ice on board, and frozen until processing. Sampling of benthic organisms was authorized by the permit N 24700-1 (MMA-ICMBio) and voucher specimens are deposited at the Unicamp Zoology Museum (http://www.splink.org.br).

### Laboratory analysis

The classification of rhodolith morphology was determined by measuring the largest, intermediate, and minor axis of each nodule, which resulted in a mean nodule diameter and sphericity for each station (Bosence & Pedley, 1982). The morphological rhodolith dataset was plotted on a TRIPLOT spreadsheet developed by Graham & Midgley (2000), and drawn on the pebble shape diagram of Sneed & Folk (1958) that discriminates rhodoliths in spheroidal, discoidal, or ellipsoidal shape. The rhodolith bed density was estimated from the number of nodules sampled within each quadrat (nodules·m^−2^). The mean rhodoliths diameter within each station was measured from all nodules in each replicate and averaged per station from the three replicated samples.

The ramification of rhodolith nodules were determined semi-qualitatively from each site based on their relative branching density (Bosence, 1983). Nodules were classified into four groups: I = a single branch; II = few branches; III = frequent branching; IV = dense and solid branching. The average internal volume of the rhodoliths in each site was determined by water volumetric displacement (Steller et al., 2003). Rhodoliths were covered with a plastic film and then submerged in a graduated container of a known volume. Later, rhodoliths were again submerged but without the plastic film. The difference between the initial volume (rhodoliths with film) and the displacement of the liquid with rhodoliths without the film was considered the rhodolith’s internal volume.

In the laboratory, rhodolith nodules were broken and epifaunal organisms were sieved (500 μm) and preserved in 70% ethanol until sorting. All organisms were identified to family or the lowest possible taxonomic level under a stereomicroscope. Macrofaunal trophic group analysis followed the main feeding types including deposit feeders, detritivores, suspension feeders, filter feeders, and omnivores, carnivores or others (OCO) according to Arruda, Domaneschi & Amaral, 2003, Jumars, Dorgan & Lindsay (2015), Queirós et al. (2013) and Macdonald et al. (2010).

For sediment grain size, carbonate content, and organic matter analysis, the samples were thawed and placed in an oven at 60 °C for 48 h. The dry sediment was macerated and taken to a stirrer, where the grain size was determined by sieving it between −1.5 phi (Φ) sieves and 4 Φ, with 1 Φ intervals. Subsequently, the values of Φ were added to the SysGran 3.0 software (Camargo, 2006) to analyze the granulometric properties (*i.e*. average grain size and the total percentage of gravel, sand, silt, and carbonate). The carbonate contents of the sediment were determined by combustion in a muffle (550 °C for 4 h) with an additional hour at 800 °C. The sediment organic matter content was determined by combustion in a muffle (550 °C for 4 h). Due to sample loss, sedimentary analysis was not done in station L3. All sedimentary organic biopolymers (carbohydrates, lipids, and proteins) analysis were made in triplicates, following the methods in Danovaro (2010). Total protein analysis (PRT) was carried after its extraction with NaOH (0.5 M, 4 h) and was determined according to Hartree (1972), modified by Rice (1982), to compensate for phenol interference. Total carbohydrate (CHO) was analyzed according to Gerchacov & Hatcher (1972). Total lipids (LPD) were analyzed according to the protocol described in Marsh & Weinstein (1966), being extracted from 1 g of homogenized sediment lyophilized by 11 ultrasonication (20 min) in 10 ml of chloroform:methanol (2:0 1 v/v). Blanks for each analysis were taken with pre-combusted sediments at 450 °C and 480 °C for 4 h. The concentrations of total protein, carbohydrate, and lipid were displayed as bovine serum albumin (BSA), glucose, and tripalmitin equivalents, respectively. The concentrations of total protein, carbohydrate, and lipid were converted into carbon equivalents assuming a conversion factor of 0.49, 0.40, and 0.75, respectively (Fabiano & Danovaro, 1994). Also, protein to carbohydrate (PRT:CHO) and carbohydrate to lipid (CHO:LPD) ratios were applied to assess the state of biochemical degradation processes (Galois et al., 2000). The sum of biopolymer concentrations was added to the analysis as a measure of biopolymeric carbon (BPC; Fabiano, Danovaro & Fraschetti, 1995; Hadlich et al., 2018).

### Statistical analysis

Benthic assemblages were compared across rhodolith beds with high and low densities (bed), and between rhodoliths and sediments (habitats). Within this sampling design we compared the nodule’s epifauna with the infaunal macrofauna of the underlying soft sediments. Macrofaunal density and diversity (alpha, gamma, and beta) were compared across bed (high and low density) and habitat (nodule and sediments) levels. We used the approach of additive partitioning to estimate alpha (α) and gamma (γ) species richness based on the sum of richness from each sample (α) within each habitat (Josefson, 2009). In addition, the macrofaunal trophic diversity was determined based on the classification of species according to their feeding modes (see Laboratory analysis description) and their density. This matrix was used to calculate the assemblage functional richness (FRic), functional dispersion (FDis), functional evenness (FEve), and entropy (FRaoQ; Mason et al., 2005); which were also tested over the spatial scales above. FRic indicates the amount of niche space (feeding mode category) filled by species in the community, FEve describes the evenness of functional distribution in a the trait space, and FDis and RaoQ are indices quantifying how functionally similar is the community spatially (Botta-Dukát, 2005; Mason et al., 2005).

Spatial differences in rhodolith bed structure (nodule density, internal volume, morphology and ramification) as well as in sediment parameters (total organic matter, carbonate, biopolymers, and granulometry) were also compared across bed and habitat levels. Spatial analyses for bed structure, sediments, and macrofaunal assemblages were made either through analysis of variance (ANOVA; Underwood, 1997) for univariate parameters or by a permutational multivariate analysis of variance (PERMANOVA; Anderson, 2017) for multivariate parameters. These tests were hierarchically designed with one fixed factor (beds, two levels: high and low); or two fixed factors, adding station (nested in bed, three levels: stations 1, 2, and 3) or habitat (fixed, two levels: rhodoliths and sediment). PERMANOVAs were based on a Bray–Curtis resemblance matrix under a reduced residuals model and data was square-root transformed to give more weight to rare taxa in the analyses (Clarke & Gorley, 2006). Post-hoc pairwise tests (Tukey for ANOVAs or PERMANOVA pairwise) were performed to identify significant differences within factor levels (Underwood, 1997; Anderson, 2008).

Macrofaunal assemblage composition was also tested based on the total dissimilarity (*i.e*. beta diversity) between beds and habitats. Dissimilarity levels were calculated from both species’ turnover (βSIM; total replacement of species) and nestedness (subsets of species among sites, βSN; Baselga, 2010), based on macrofaunal presence-absence data (Sørensen index). A cluster dendrogram was also applied using the average abundance of all taxa from the Bray-Curtis similarity matrix. Additionally to that, a non-metric Multidimensional Scaling Analysis (nMDS) was applied to visualize the similarities of macrofauna assemblies between densities and habitats, using the square-root abundance of all taxa from the Bray-Curtis similarity matrix. A canonical analysis of principal coordinates (CAP; Anderson & Willis, 2003) was performed to determine the association between environmental variables and benthic assemblages between beds and habitats. Graphic design and analysis were performed using R Project (R Core Team, 2014) with packages: ‘ggplot2’ (Wickham, 2016), ‘oce’ (Kelley & Richards, 2017), ‘stats’, ‘vegan’ (Oksanen et al., 2018), MASS (Ripley et al., 2019), mgcv (Wood, 2012), MuMIn (Barton & Barton, 2013), FD (Laliberté, Legendre & Shipley, 2014), and ‘ggdendro’ (Andrie & Ripley, 2020). Macrofaunal raw data is openly available through the Ocean Biogeographic Information System (OBIS) portal (Stelzer et al., 2021).

## Results

### Rhodolith bed characteristics

The maximum depth of sampling stations varied between 39 and 55 m and did not differ significantly between rhodolith beds of high and low density (Table 1). During sampling, surface and bottom temperature ranged from 26–28 °C and 19–23 °C, respectively, while salinity ranged from 37.7–38.3 (Table 1; Fig. S1). Stations H1 and H2 had water column profiles with a marked halocline in the first 10 m, whereas at stations H3, L2, and L3 the halocline occurred at 15 to 35m depth. Temperature showed a similar bathymetric profile between stations. Secchi’s depth varied between 20–35 m deep and the incidence of light in the water column reached greater depths in station L2 (Table 1).

**Figure 3:**
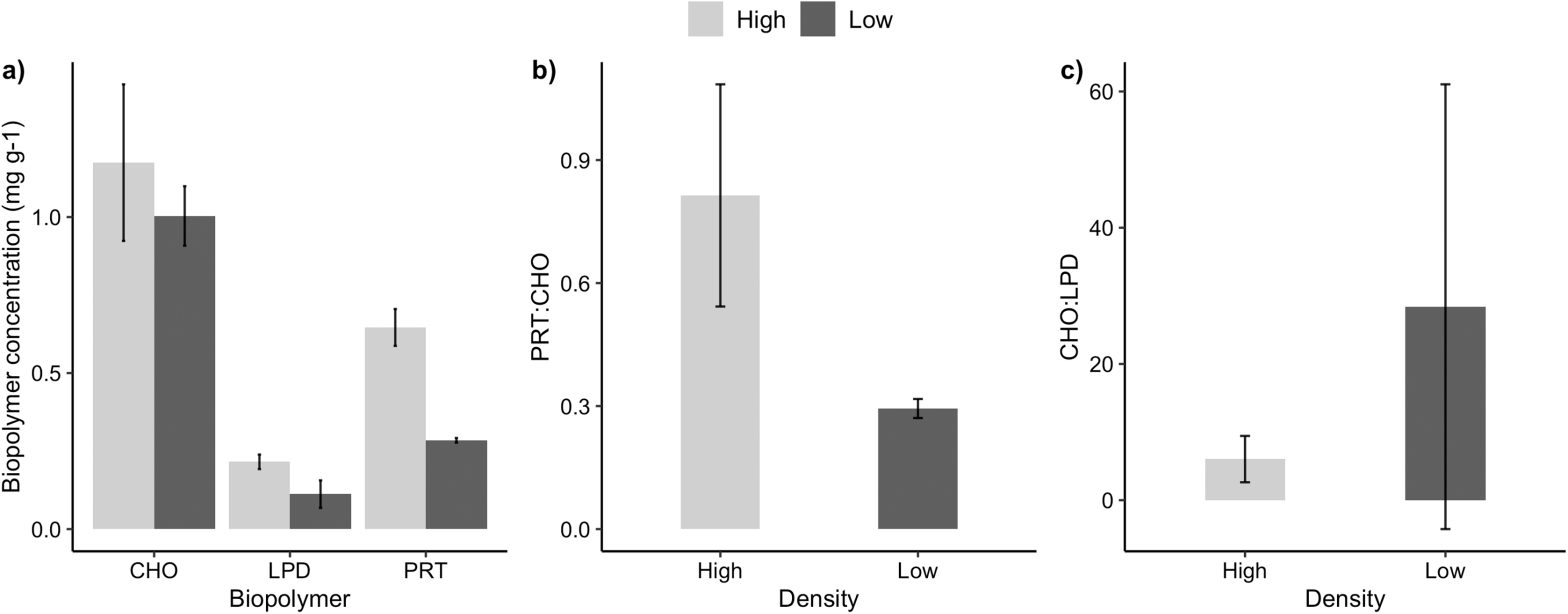
Sediment biopolymer concentrations. Average (±SE) sediment biopolymer concentrations under rhodolith beds of high and low density. (A) Carbohydrate (CHO), lipids (LPD), and proteins (PRT), (B) PRT:CHO ratio, and (C) CHO:LPD ratio.

The sediments under the high and low-density beds had a similar grain size composition with predominance of coarse and medium sand (F = 3.51, *p* = 0.07; Table 2). The percentage of sediment total organic matter varied between 2% and 5%, with no significant differences between high and low-density beds (F = 0.038, *p* = 0.849). Sediment carbonate content ranged from 3% and 8%, with higher carbonate content under the high-density beds (F = 5.74, *p* = 0.0323). Two sediment organic biopolymers (proteins and lipids) had higher concentrations in high-density beds (F = 27.3, *p* = 0.0002; Table 2, Fig. 3), whereas carbohydrate concentrations were similar. Protein (PRT) and lipid concentrations in sediments of high-density beds ranged from 0.43 to 0.85 mg·g^−1^ and 0.02 to 0.30 mg·g^−1^, respectively (Fig. 3). The total biopolymeric carbon and biopolymer ratios (protein: carbohydrate, carbohydrate:lipid) did not differ at the scale of beds, and showed a wide range among samples (0.23 to 2.81 protein:carbohydrate, 2.4 to 111.3 carbohydrate:lipid). Biopolymeric carbon concentration did not vary in sediments under rhodolith beds.

**Table 2:** ANOVA and PERMANOVA results for sedimentary data.

	*df*	TOM^1^				Carbonate^1^				Grain size^2^					BPC^1^							
		SS	MS	F	*p*	SS	MS	F	*p*	SS	MS	F	R^2^	*p*	*SS*	*MS*	*F*	*p*				
Beds	1	0.03	0.03	0.038	0.849	5.76	5.76	5.74	**0.0323**	0.01	0.01	3.51	0.21	0.07	1.38	1.38	4.15	0.0643				
Residuals	13	9.97	0.77			13.05	1.00			0.05	0.004		0.79		4.01	0.33						
Total	14									0.06			1.00									
	***df***	**PRT^1^**				**CHO^1^**				**LPD^1^**					**PRT:CHO^1^**				**CHO:LPD^1^**			
		**SS**	**MS**	**F**	***p***	**SS**	**MS**	**F**	***p***	**SS**	**MS**	**F**	***p***		**SS**	**MS**	**F**	***p***	**SS**	**MS**	**F**	***p***
Beds	1	0.44	0.44	27.3	**0.0002**	0.10	0.10	0.31	0.584	0.03	0.03	4.95	**0.0459**		1.14	1.14	2.71	0.126	2,781	2,781.4	3.84	0.0735
Residuals	12	0.19	0.01			3.79	0.31			0.09	0.01				5.06	0.42			8,682	723.5		

**Note:**

Results of ANOVA^1^ and PERMANOVA^2^ tests comparing spatial differences of sediment variables across rhodolith beds (high and low density). TOM, total organic matter; BPC, biopolymeric carbon; PRT, proteins; CHO, carbohydrates; LPD, lipids; and their ratios (PRT/CHO and CHO/LPD). Significant results (*p* < 0.05) are in bold. *df*, degrees of freedom; SS, sum of squares; MS, mean square; F and *p* statistics.

**Figure 4:**
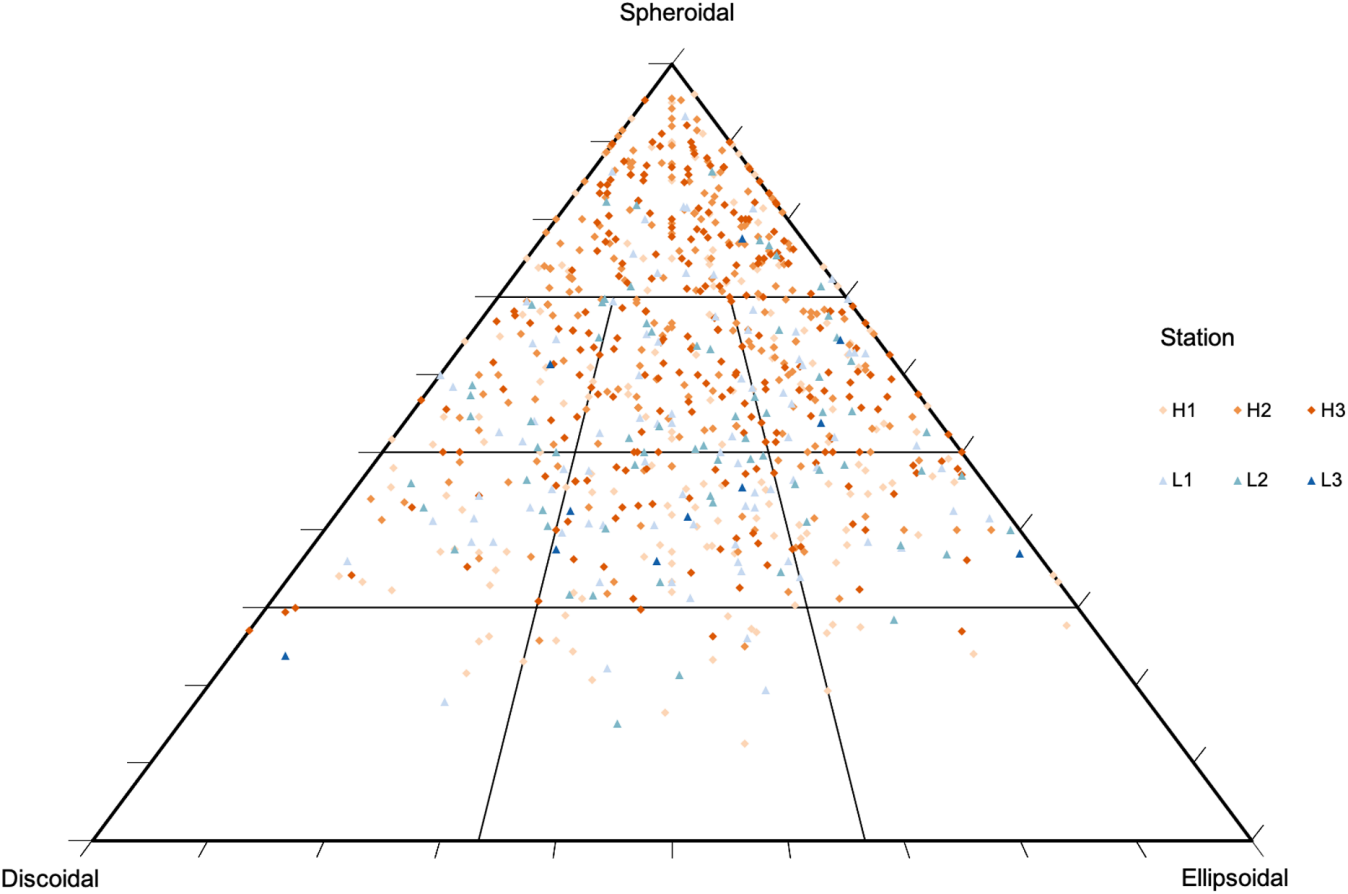
Rhodolith nodule morphology. Morphological distribution of rhodolith nodules sampled in this study on a TRIPLOT diagram (Graham & Midgley, 2000; Sneed & Folk, 1958). Warm colors represent nodules from high-density beds and cold colors represent nodules from low-density beds.

Rhodolith nodule density in high-density beds (204 ± 18.7 m^−2^) was over 3 times higher when compared to the low-density beds (61 ± 27.1 m^−2^; F = 47.9, *p* < 0.0001; Table 3). High-density beds were dominated by rounded (36%) and elongated (15%) nodules, whereas in low-density beds the nodules were predominantly discoidal (22%) and spherical (14%; F = 3.12, *p* = 0.05; Table 3, Fig. 4). Overall, nodule mean diameter and the ramification pattern were similar between high-density and low-density beds, with small nodules (3.7 to 5.3 cm mean diameter) with dense and solid branching (88% of ramification type IV; *p* < 0.01; Tables 1 and 3). An exception of this patter was observed at H1 where nodule diameter was significantly lower than at the other stations (F = 7.69, *p* = 0.0071, F = 5.17, *p* = 0.05; Tables 1 and 3). The diameter of rhodolith nodules at low-density beds were more heterogeneous than at the high-density beds (F = 7.69, *p* = 0.0071; Table 3). The average rhodolith nodule internal volume in high-density beds (312.2 cm^3^·m^−2^) was similar with the low-density beds (206.3 cm^3^·m^−2^), but with local differences between stations H3 and L3 (F = 5.68, *p* = 0.0183; Table 3).

**Table 3:** ANOVA and PERMANOVA results for rhodolith data.

	*df*	Density^1^				Internal Volume^1^				Diameter^1^			
		SS	MS	F	*p*	SS	MS	F	*p*	SS	MS	F	*p*
Bed	1	10,272.2	10,272.2	47.9	**<0.001**	36,630	36,630	2.07	0.175	3.91	3.91	48.57	**<0.001**
St(Bed)	2	84.1	42.1	0.2	0.8242	79,295	39,648	2.24	0.1480	4.98	2.49	30.93	**<0.001**
Interaction Bed*St	2	2,087.4	1,043.7	4.8	**0.0282**	2,000,249	100,124	5.68	**0.0183**	1.24	0.62	7.69	**0.0071**
Residuals	12	2,569.3	214.1			211,521	17,627			0.96	0.08		
Tukey post-hoc	**H1 ≠ L3** **H2 ≠ L2, L3** **H3 ≠ L1, L2, L3**					**H3 ≠ L3**				**H1 ≠ H2, H3, L1, L2, L3** **L1 ≠ L2 ≠ L3** **H3 ≠ L2**			
	***df***	**Morphology^2^**				**Ramification^2^**							
		**SS**	**MS**	**F**	***p***	**SS**	**MS**	**F**	***p***				
Bed	1	0.24	0.24	3.12	**0.05**	0.02	0.02	1.32	0.33				
St(Bed)	1	0.13	0.13	1.76	0.10	0.06	0.06	5.11	**0.03**				
Interaction Bed*St	1	0.15	0.15	1.89	0.15	0.07	0.07	5.17	**0.05**				
Residuals	14	1.11	0.08			0.18	0.01						
Total	17	1.64				0.32							
PERMANOVA pairwise results						ns							

**Note:**

Results of ANOVA^1^ and PERMANOVA^2^ tests of rhodolith nodule Density, Internal Volume, mean diameter (Diameter), Morphology and Ramification across beds of high and low density (Bed), and stations (St). Significant results (*p* < 0.05) are in bold. *df*, degrees of freedom; SS, sum of squares; MS, mean square; F and *p* statistics, ns, not significant post-hoc tests.

### Macrofaunal assemblages

**Figure 5:**
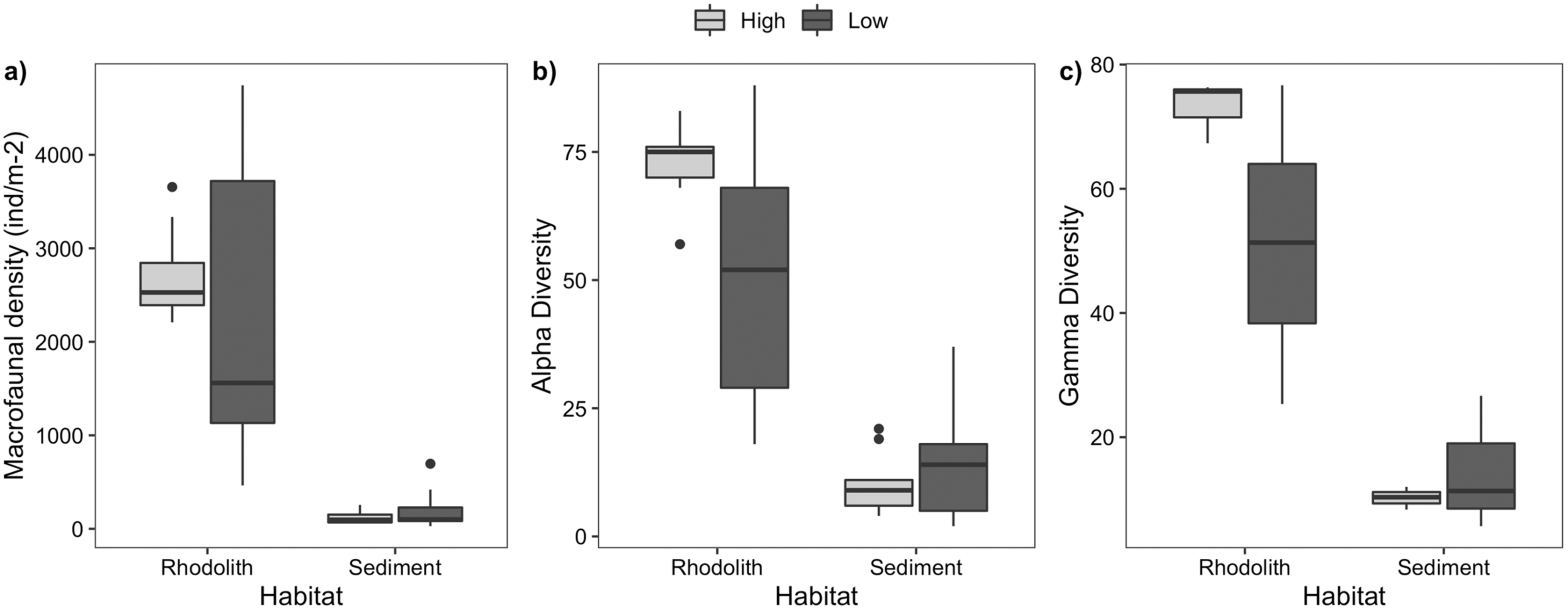
Macrofaunal abundance and richness. Macrofaunal structure in rhodoliths and sediments at high- and low-density stations. (A) Macrofaunal density (ind·m^−2^), (B) alpha diversity, and (C) gamma diversity.

We sampled a total of 11,421 macrofaunal organisms associated with the rhodolith beds (epifauna) and underlying sediments (infauna), and registered significant differences between high and low-density beds and habitats (rhodoliths and sediment). Rhodolith nodules in high-density beds had a similar macrofaunal density (2,736 ± 161.7 ind·m^−2^) when compared to low-density beds (2,339 ± 554.9 ind·m^−2^; F = 0.27, *p* = 0.601; Table S1, Fig. 5); but in both cases, macrofaunal density was significantly higher on nodules (2,538 ± 288.7 ind·m^−2^) when compared to the sediments below nodules (166 ± 38.8 ind·m^−2^; F = 66.18, *p* < 0.0001). We identified 151 different macrofaunal taxa within nodules and sediments, with pronounced contrasts between both habitats. There was a higher alpha and gamma diversity in rhodoliths when compared to the sediment infauna (F = 38.6, *p* = 0.0002).

Macrofaunal composition changed markedly at the scale of beds (F= 3.41, *p* = 0.02) and between habitats (F = 20.0, *p* = 0.01; Table S1). Rhodolith nodules were mostly dominated by Annelida (47%) and Crustacea (44%) with a marked difference between high and low-density beds. Annelida (Syllidae, Nereididae, and *Lysidice* sp) dominated high-density beds (51%), whereas Crustacea (Gammaridae, Melitidae, and *Elasmopus* sp) dominated (64%) low-density beds (Fig. S2). In contrast, sediment macrofaunal assemblages were relatively similar under high and low-density beds being dominated by Crustacea (63%; Ostracoda, Melitidae, and *Leptochelia* sp) and Mollusca (22%; *Meioceras* sp and Cardiidae; Fig. S2).

**Figure 6:**
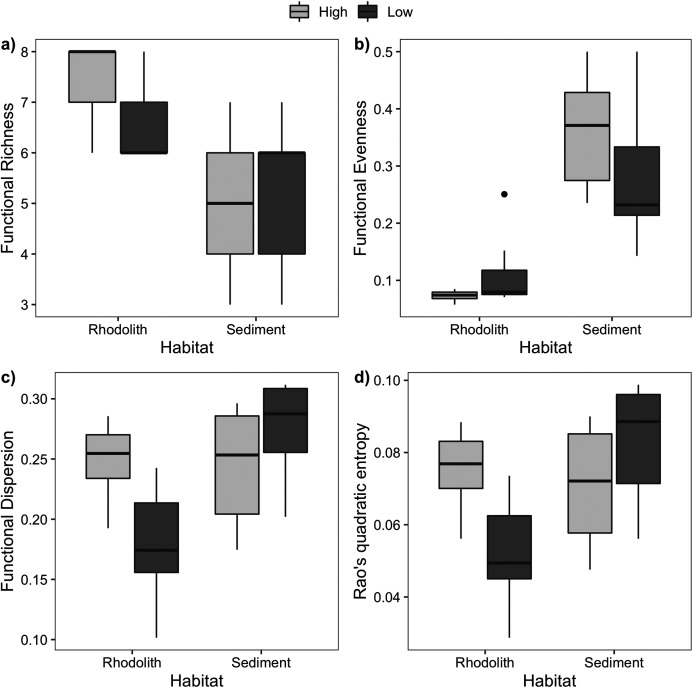
Macrofaunal functional diversity. Mean (±SE) macrofaunal functional diversity indices across high and low-density rhodolith beds. (A) Functional richness (FRic), (B) functional evenness (FEve), (C) functional dispersion (FDis), and (D) entropy (Rao Q).

Macrofaunal trophic richness exhibited a distinct dominance between habitats (Fig. S2). The dominant trophic groups on the rhodolith nodules were omnivores, carnivores, and others feeders (OCO, 74.6%), while sediments were dominated by filter feeders (38.5%) and OCO (28.5%). Macrofaunal trophic richness (FRic) was higher on the rhodolith nodules (7 ± 0.2), when compared to the sediment underneath (5 ± 0.4; F = 27, *p* < 0.0001; Fig. 6). Sediments presented a more homogeneous trophic evenness (FEve = 0.32 ± 0.03) when compared to the rhodolith nodules (FEve = 0.09 ± 0.02; F = 75.39, *p* < 0.0001; Table S2). Macrofaunal trophic dispersion (FDis) and entropy (FRaoQ) differed between habitats only at low-density beds, which were both higher in sediments (Table S2; Fig. 6).

**Figure 7:**
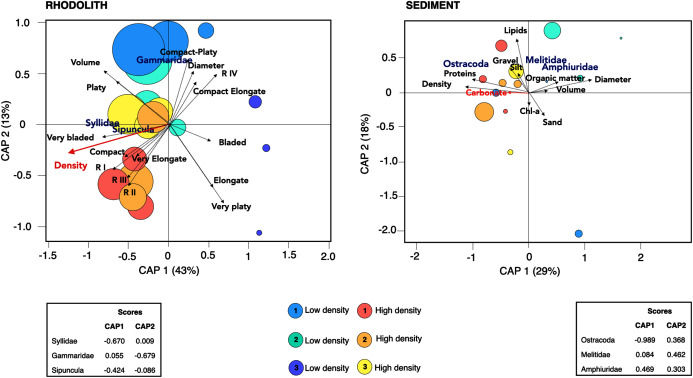
Canonical analysis of principal coordinates. Canonical analyses of principal coordinates (CAP) indicating differences in the macrofaunal assemblages in rhodolith beds and the underlying sediments at high density (H1, H2, H3; warm colors) and low-density (L1, L2 and L3; cold colors) beds. Vectors are based on Spearman correlation values > 0.5 (*p* < 0.5) for environmental variables and scores for each taxa. The proportion of data explained by axis 1 and 2 are in parenthesis. Size of circles represents macrofaunal total abundance in each station.

There was a high degree of patchiness with high dissimilarity among macrofaunal assemblages between rhodolith nodules and sediments (Sørensen Index = 0.92; Fig. S3). Macrofaunal composition in rhodoliths and underlying sediments was marked by low nestedness and high taxa turnover (Table 4). The CAP analysis supported the spatial dissimilarity in macrofaunal assemblages inside nodules, suggesting a strong effect of bed density on nodule’s macrofaunal density (F = 7.97, *p* = 0.002; Fig. 7, Table S3). On the other hand, the sediment macroinfauna under rhodolith beds were significantly related to sediment carbonate content (F = 1.71, *p* < 0.01; Table S4, Fig. 7). The CAP ordination showed that bed density influenced the rhodolith macrofaunal composition with greater contribution of Syllidae and Sipuncula in high-density beds, whereas low-density beds were associated with gammarid amphipods (Fig. 7). The underlying sediments at high-density beds were predominantly dominated by ostracods, but crustaceans Melitidae and Amphiuridae dominated at low-density beds (axis 1; >29%).

**Table 4:** Macrofaunal dissimilarity, nestedness and turnover.

	Dissimilarity indices				
	Sørensen	βSIM	βSNE	C-score	Pr(sim)
				(species mean)	
Rhodolith + Sediment	0.92	0.77	0.14	12.58	**0.01**
Rhodolith	0.88	0.80	0.08	5.49	**0.01**
Sediment	0.89	0.80	0.09	0.84	0.99

**Note:**

Dissimilarity (Sørensen index), nestedness (βSIM) and turnover (βSNE) in the benthic macrofaunal assemblages in rhodolith nodules and sediments. Significant results are in bold.

## Discussion

Rhodolith beds are unique marine habitats and are expected to increase the seafloor biodiversity by increasing its structural complexity and organic input. Our study support that rhodolith beds have a higher macrofaunal abundance and are home to a more diverse and distinct set of benthic taxa when compared to the underlying sediments, supporting our first hypothesis that these habitats may increase local and regional biodiversity. Our results are thus similar to previous assessments of the effects of rhodolith beds on benthic assemblages (Carvalho, Loiola & Barros, 2017; Gabara et al., 2018), and reveal similar ecological processes of substrate complexity governing benthic ecosystems observed in other coastal marine habitats (Archambault & Bourget, 1996; Mazzuco, Stelzer & Bernardino, 2020). We also observed that spatial heterogeneity in rhodolith nodules density and in the underlying sediments contribute to maintaining a greater species diversity and a higher dissimilarity in species composition between rhodolith and sedimentary habitats. These changes are based on a high species turnover between rhodoliths and sediments, revealing that the sediment macrofauna under rhodoliths is not a subset of species inhabiting rhodolith nodules (and vice versa). Our findings also suggest a potential trophic link between rhodolith nodules and the macrofaunal assemblages in underlying unconsolidated sediments, through changes in sediment carbonate and organic contents. In addition, through an increased deposition of organic matter under high-density beds, rhodoliths may have positive effects on carbon burial in sediments of continental shelves that are yet poorly quantified.

In our study, we have detected significant differences in nodule’s diameter, internal volume and ramification between sampled stations (scale of 10–100’m), suggesting a marked regional variability in bed structure. Rhodoliths showed a predominant elongated morphology in high-density beds likely due to a greater bed stability (Hinojosa-Arango & Riosmena-Rodríguez, 2004; Gagnon, Matheson & Stapleton, 2012), whereas low-density beds had a predominance of compact nodules. Our marked regional variability in rhodolith nodule structure thus suggests more complexity over rhodolith beds than anticipated previously. Large-scale patterns in the transition between tropical-temperate rhodolith beds in Brazil have been described with tropical areas holding high-density beds with a smaller mean diameter when compared to low latitude regions (Amado-Filho et al., 2007; Amado-Fiho et al., 2017; Riul et al., 2009). Our study has a clear limited latitudinal sampling, but evidences that the structure of beds is highly variable at the scale of 10’s of meters. This high spatial heterogeneity was also observed in beds with lower density of nodules. The morphological variability in nodules may thus provide more internal space for the colonization of macrofauna, likely increasing macrofaunal density and diversity through higher niche availability when compared to underlying sediments. There are also morphological changes in the form and sphericity of rhodolith nodules along depth ranges over larger spatial scales (Otero-Ferrer et al., 2020; Holz et al., 2020; Veras et al., 2020). This study sampled beds over a depth range of 30 to 60 m, so depth ranges may also be associated to the morphological variability in nodules. In addition, although we did not identify macroalgal diversity and rhodolith forming species to determine their specific effects over the benthic macrofauna, these effects may also influence the rhodolith’s morphology and benthic ecological patterns (Foster, 2001; Hinojosa-Arango & Riosmena-Rodríguez, 2004; Anderson et al., 2021).

The hypothesis of the effects of rhodolith nodule’s density on benthic macrofaunal assemblages was partially supported by the higher density and different composition of taxa in high-density beds. These effects were similar to those observed within rhodolith beds in the Mediterranean and on the coast of California (Steller et al., 2003; Hinojosa-Arango & Riosmena-Rodríguez, 2004), suggesting that the abundance of rhodolith nodules is key to both regional and large-scale patterns of benthic diversity. Polychaeta and Crustacea dominated beds in SE Brazil in a similar pattern observed in other continental margins at similar depths (Bordehore, Ramos-Esplá & Riosmena-Rodríguez, 2003; Hinojosa-Arango & Riosmena-Rodríguez, 2004; Grall et al., 2006; Harvey & Bird, 2008). High-density beds were dominated by Polychaeta, especially Syllidae; while low-density beds were dominated by Crustacea, mostly Gammaridae. The greater abundance and dominance of these groups in rhodoliths may be related to their wide feeding strategies, favoring the use of diverse microhabitats (Harvey & Bird, 2008; Sciberras et al., 2009). Macrofaunal composition was significantly related to bed density and between rhodolith or sedimentary habitats. Although we observed a marked small-scale patchiness in rhodolith morphology, ramification, diameter and volume, macrofaunal composition were unrelated to these effects in our study site. Other studies have detected associations between benthic assemblages and rhodoliths structure (Steller et al., 2003; Sciberras et al., 2009; Tompkins & Steller, 2016). Our data suggests that these effects are at scales of individual stations, possibility reflecting a stronger spatial variability within our study area that needs to be considered. It is also likely that temporal changes in macrofaunal structure occur within these dynamic ecosystems as a result of bottom transport and disturbance (Navarro-Mayoral et al., 2020), which will need to be assessed for our study area.

Our study revealed that underlying sediments in rhodolith beds support a distinct set of macrofaunal organisms and with lower alpha and gamma diversity when compared to the nodules. Mollusks and crustaceans were the most dominant groups in the sediment. Most of them were small species with a predominant burrowing behavior, which favors life in unconsolidated habitats and were also reported at other rhodolith beds (Snelglove & Buttman, 1995; De Grave, 1999). Carbonate also played an important role in structuring sediment macrofaunal assemblages at high-density beds, probably due to the greater aggregation and local input of carbonate from erosion of nodules. This finding confirms that rhodoliths have a strong influence on sediment macrofaunal structure by changing their sedimentary habitat (De Grave, 1999), and to a lesser extent their organic content. The organic supply from rhodoliths to underlying sediments is supported by a higher concentration of proteins in high-density stations, even though these changes were not related to macrofaunal composition. The higher organic matter quality, rich in proteins and lipids, may come from a higher pelagic detrital input being deposited, and also from an increased algal input from rhodoliths in high-density nodules (Grall et al., 2006). These results suggest that the physical structure of rhodoliths may be very important for the organic input to the benthos on the nodules and in the underlying sediments. These differences indicate that the macrofaunal composition and diversity within rhodolith beds are closely linked to food availability (Grall et al., 2006). The type and abundance of food items may support the observed dominance of omnivore and carnivore feeders in rhodolith beds, in contrast to the predominance of suspension feeders in sediments under the nodules. Trophic group richness was similar between rhodolith beds and the underlying sediments, suggesting a wide niche availability in both habitats (Paganelli, Marchini & Occhipinti-Ambrogi, 2012; Bolam et al., 2017).

Macrofaunal functional richness, evenness, dispersion, and entropy were markedly distinct between the rhodoliths and sediments, but with marked small-scale patchiness within beds. We observed an increased functional richness in rhodolith nodules when compared to sediments independently of nodule density, suggesting that functional richness is strongly increased with the presence of rhodoliths on the seafloor, even at low nodule densities. This view is further supported by the distinct set of taxa and higher taxon richness in rhodolith beds, which were markedly dissimilar from the underlying sediment habitats. As a result of higher taxon richness, macrofaunal assemblages associated with the rhodoliths were more functionally heterogeneous. These patterns suggest an increased niche availability associated with these biogenic structures, which support a higher number of taxa with unique ecological functions on the rhodolith nodules (Mason et al., 2005; Schumm et al., 2019).

The marked differences in macrofaunal density, composition and diversity (taxonomic and functional) within rhodolith beds support their value as biodiversity hotspots along continental shelves. The strong effect of bed density in macrofaunal assemblages in nodules and in sediments, suggest that the physical structure of rhodolith beds is important by providing habitat and organic input to the benthos. As a result, anthropogenic impacts that may influence the habitat structure of beds through physical disturbance, including fishing and dredging, pose a high threat to their biodiversity (Hall-Spencer & Moore, 2000; Grall & Hall-Spencer, 2003). In SE Brazil, multiple uses and impacts such as fishing, coastal urbanization (*e.g*. ports, marinas), pollution and mine tailings (Gomes et al., 2017; Vilar et al., 2020), are of additional concern to rhodolith beds in areas that may receive increased sediment deposition and contamination. This study supports those initiatives that aim to preserve and manage these ecosystems on the Brazilian continental shelf will lead to the protection of a larger number of species. According to our results, physical impacts that lead to the removal or burial of rhodolith nodules will cause a marked loss of species and their functional diversity, with potential implications to fisheries and to other species that rely on these ecosystems for habitat or food. As Brazil holds extensive rhodolith areas, setting priority areas for conservation in areas of higher nodule density could protect assemblages with higher functional diversity and thus more resilient to local and climate stressors, with additional potential carbon burial benefits.

## Conclusions

This study evidences that rhodoliths provide a unique habitat for a diverse (taxonomically and functionally) and distinct benthic assemblage, with 5 to 7 times more species and 10 times more macrofaunal organisms when compared to the underlying sediments. According to our findings, the presence and higher density of rhodolith nodules are key to benthic macrofaunal assemblages, likely due to increased niche availability and increased organic input when compared to sediments underlying those beds. Rhodolith beds sustained higher concentrations of carbonate, proteins, and lipids, providing a high food quality habitat to benthic assemblages. Considering that rhodolith beds are vulnerable to global changes and the exploratory pressure grows upon these habitats, our results support the importance of these ecosystems to overall marine biodiversity on the Brazilian continental margin, and we recommend improved restrictions to preserve these habitats.

## Supplemental Information

10.7717/peerj.11903/supp-1 Supplemental Information 1 Raw data. The presence/absence of benthic organisms in each sample, and the statistical results not included in the main text.

10.7717/peerj.11903/supp-2 Supplemental Information 2 Supplemental Figures and Tables.

## Additional Information and Declarations

### Field Study Permissions

The following information was supplied relating to field study approvals (*i.e*. approving body and any reference numbers): Field sampling was approved by the Brazilian Ministry of Environment (ICMBio) through a license in name of Angelo Bernardino (license number 24700-1).

### Data Availability

The following information was supplied regarding data availability: The raw data are available in the Supplemental Files.
