# Supplementary material for: Taxonomic and functional diversity of benthic macrofauna associated with rhodolith beds in SE Brazil
Source: PeerJ. 2021 Jul 29;9:e11903. doi: 10.7717/peerj.11903 (PMC8325915; doi:10.7717/peerj.11903)
Supplement: Supplemental Information 2 [file peerj-09-11903-s002.docx]

**Taxonomic and functional diversity of benthic macrofauna associated with rhodolith beds in SE Brazil, by Patricia Stelzer, et al., PeerJ 2021**

**Supplementary tables and figures**

**Table S1.** Results of ANOVA^1^ and PERMANOVA^2^ , and significant post-hoc tests comparing macrofaunal structure across rhodolith beds (high and low density) and habitats (nodules or sediments). Significant results (*p* < 0.05) are in bold. Note: *df* degrees of freedom, SS sum of squares, MS mean square, F and *p* statistics.

|  | Macrofaunal Density1 | | | | |  |  | Alpha Diversity | | | | |
| --- | --- | --- | --- | --- | --- | --- | --- | --- | --- | --- | --- | --- |
|  | *df* | SS | MS | F | *p* |  |  | *df* | SS | MS | F | *p* |
| Beds (B) | 1 | 21x10^4^ | 21x10^4^ | 0.27 | 0.601 |  | Beds (B) | 1 | 702 | 702 | 3.49 | 0.0708 |
| Habitat (H) | 1 | 51x10^6^ | 51x10^6^ | 66.18 | **< 0.0001** |  | Habitat (H) | 1 | 22251 | 22251 | 110.70 | **< 0.0001** |
| B*H | 1 | 53x10^4^ | 53x10^4^ | 0.69 | 0.410 |  | B*H | 1 | 1560 | 1560 | 7.71 | **0.0089** |
| Residuals | 32 | 24x10^6^ | 76x10^4^ |  |  |  | Residuals | 32 | 6434 | 201 |  |  |
|  |  |  |  |  |  |  | TukeyHSD significant results: | Low-density beds (nodules) ≠ High-density beds (nodules)  Low and high-density beds (nodules) ≠ (sediments) | | | | |
|  | Gamma Diversity | | | | |  |  |  | Macrofauna assemblage composition^2^ | | | |
|  | *df* | SS | MS | F | *p* |  |  | *df* | SS | MS | F | *p* |
| Beds (B) | 1 | 225 | 225 | 0.66 | 0.4381 |  | Beds (B) | 1 | 0.59 | 0.59 | 3.41 | **0.0200** |
| Habitat (H) | 1 | 13068 | 13068 | 38.61 | **0.0002** |  | Habitat (H) | 1 | 3.51 | 3.51 | 20.01 | **0.0100** |
| B*H | 1 | 800 | 800 | 2.34 | 0.1626 |  | B*H | 1 | 0.46 | 0.46 | 2.63 | 0.0600 |
| Residuals | 32 | 2708 | 339 |  |  |  | Residuals | 32 | 5.61 | 0.17 |  |  |
|  |  |  |  |  |  |  | Total | 35 | 10.18 |  |  |  |

**Table S2.** Results of ANOVA and significant post-hoc tests on macrofaunal functional diversity among rhodolith beds (bed, two levels, high and low density) and habitats (nodules and sediments). FRic – functional richness, FDis – functional dispersion, FEve- functional evenness, and FRaoQ- functional entropy. Significant results (*p* < 0.05) in bold. Note: *df* degrees of freedom, SS sum of squares, MS mean square, F and *p* statistics.

|  |  | FRic | | | |  |  |  | FEve | | | |
| --- | --- | --- | --- | --- | --- | --- | --- | --- | --- | --- | --- | --- |
|  | df | SS | MS | F | *p* |  |  | *df* | SS | MS | F | *p* |
| Beds (B) | 1 | 0.44 | 0.44 | 0.33 | 0.568 |  | Beds (B) | 1 | 0.004 | 0.004 | 0.69 | 0.4114 |
| Habitat (H) | 1 | 36.00 | 36.00 | 27.00 | **< 0.0001** |  | Habitat (H) | 1 | 0.47 | 0.47 | 75.39 | **< 0.0001** |
| B*H | 1 | 2.78 | 2.78 | 2.08 | 0.159 |  | B*H | 1 | 0.03 | 0.03 | 5.26 | **0.0284** |
| Residuals | 32 | 42.67 | 1.33 |  |  |  | Residuals | 32 | 0.20 | 0.006 |  |  |
|  |  | | | | |  | TukeyHSD significant results: | Rhodolith High ≠ Sediment High, Low  Rhodolith Low ≠ Sediment High, Low | | | | |
|  |  | FDis | | | |  |  |  | FRaoQ | | | |
|  | df | SS | MS | F | *p* |  |  | *df* | SS | MS | F | *p* |
| Beds (B) | 1 | 0.003 | 0.003 | 1.88 | 0.1789 |  | Beds (B) | 1 | 0.0002 | 0.0002 | 1.21 | 0.2793 |
| Habitat (H) | 1 | 0.01 | 0.01 | 9.61 | **0.004** |  | Habitat (H) | 1 | 0.001 | 0.001 | 6.56 | **0.0152** |
| B*H | 1 | 0.02 | 0.02 | 13.40 | **0.0008** |  | B*H | 1 | 0.002 | 0.002 | 13.85 | **0.0007** |
| Residuals | 32 | 0.05 | 0.001 |  |  |  | Residuals | 32 | 0.006 | 0.0002 |  |  |
| TukeyHSD significant results: | Rhodolith High ≠ Sediment Low  Rhodolith Low ≠ Sediment High, Low | | | | |  | TukeyHSD significant results: | Rhodolith Low ≠ Rhodolith High  Rhodolith Low ≠ Sediment Low | | | | |

**Table S3.** Results of canonical analyses of principal coordinates (CAP) to evaluate the contribution of rhodolith morphological parameters (density, volume, diameter) and characteristics (Compact, Compact-Platy, Compact-Elongate, Platy, Bladed, Elongate, Very platy, Very bladed, and Very elongate), and ramification (number of spikes 1 to 4) to variations in the benthic assemblage composition (abundance per taxa) in the study area. Spearman correlation values for each environmental variable are described for in CAP axis 1-2. Note: proportion of variability explained by CAP axes are between parenthesis ‘()’, F for statistic, significant results (*p* < 0.05) are in bold.

|  | F = 1.85, *p* = 0.143 | | | |
| --- | --- | --- | --- | --- |
|  | CAP 1 (43%) | CAP 2 (13%) | F | *p* |
| Density | -0.76 | -0.15 | 7.97 | **0.002** |
| Volume | -0.55 | 0.36 | 2.22 | 0.127 |
| Diameter | 0.20 | 0.36 | 1.53 | 0.264 |
| Compact | -0.34 | -0.18 | 1.84 | 0.188 |
| Compact-Platy | 0.15 | 0.45 | 1.45 | 0.273 |
| Compact-Elongate | 0.22 | 0.29 | 0.89 | 0.577 |
| Platy | -0.42 | 0.28 | 2.21 | 0.136 |
| Bladed | 0.32 | -0.08 | 1.34 | 0.338 |
| Elongate | 0.35 | -0.38 | 1.73 | 0.221 |
| Very platy | 0.43 | -0.47 | 1.50 | 0.273 |
| Very bladed | -0.54 | -0.06 | 1.37 | 0.293 |
| Very elongate | -0.30 | -0.17 | 0.61 | 0.787 |
| Number of spikes |  |  |  |  |
| 1 | -0.47 | -0.26 | 1.44 | 0.312 |
| 2 | -0.32 | -0.34 | 1.41 | 0.299 |
| 3 | -0.29 | -0.28 | 0.85 | 0.592 |
| 4 | 0.39 | 0.33 | 1.30 | 0.331 |

**Table S4.** Results of canonical analyses of principal coordinates (CAP) to evaluate the contribution of sediment composition (organic matter, carbonate, gravel, sand, silt, proteins, lipids, chlorophyll-a) and rhodolith morphological parameters (density, volume, diameter) to variations in the benthic assemblage composition (abundance per taxa) in the study area. Spearman correlation values for each environmental variable are described for in CAP axis 1-2. Note: proportion of variability explained by CAP axes are between parenthesis ‘()’, F for statistic, significant results (*p* < 0.05) are in bold.

|  | F = 1.33, *p* = 0.206 | | | |
| --- | --- | --- | --- | --- |
|  | CAP 1 (29%) | CAP 2 (18%) | F | *p* |
| Organic matter | 0.36 | 0.10 | 1.13 | 0.355 |
| Carbonate | -0.09 | -0.002 | 2.00 | **0.051** |
| Gravel | -0.19 | 0.22 | 0.93 | 0.512 |
| Sand | 0.19 | -0.23 | 0.99 | 0.445 |
| Silt | -0.13 | 0.19 | 1.25 | 0.276 |
| Proteins | -0.68 | 0.12 | 1.61 | 0.124 |
| Lipids | -0.002 | -0.13 | 1.51 | 0.185 |
| Chlorophyll-a | -0.15 | 0.53 | 1.49 | 0.151 |
| Density | -0.78 | 0.06 | 1.38 | 0.226 |
| Volume | 0.22 | 0.01 | 1.19 | 0.326 |
| Diameter | 0.77 | 0.13 | 1.20 | 0.339 |

**Table S5.** List of taxa and mean benthic macrofauna total abundance in RBs and unconsolidated sediment (±SE) through the sampled stations.

| **Taxonomic group**  **and Taxa** | **Rhodolith beds** | | | | | |  | **Unconsolidated sediment** | | | | | |
| --- | --- | --- | --- | --- | --- | --- | --- | --- | --- | --- | --- | --- | --- |
|  | H1 | H2 | H3 | L1 | L2 | L3 |  | H1 | H2 | H3 | L1 | L2 | L3 |
| **Annelida**  N id sp1 | 10.0 (± 1.2) | 8.3 (± 3.0) | 30.0 (± 5.5) | 4.7 (± 2.4) | 4.0 (± 1.2) | 1.7 (± 0.3) |  | _ | _ | _ | _ | _ | _ |
| N id sp2 | 0.7 (± 0.7) | _ | _ | _ | _ | _ |  | _ | _ | _ | _ | _ | _ |
| N id sp3 | 3.3 (± 0.3) | 4.3 (± 0.9) | 2.0 (± 0.6) | 2.0 (± 1.5) | 0.3 (± 0.3) | _ |  | _ | _ | _ | _ | _ | _ |
| N id sp4 | 1.3 (± 0.7) | 1.0 ± (0.6) | _ | _ | _ | _ |  | _ | _ | _ | _ | _ | _ |
| N id sp6 | 0.3 (± 0.3) | _ | _ | _ | 1.3 (± 1.3) | 0.7 (± 0.3) |  | _ | _ | _ | _ | _ | _ |
| N id sp7 | 10.0 (± 5.3) | 0.3 (± 0.3) | 0.3 (± 0.3) | 1.7 (± 1.2) | 1.3 (± 1.3) | _ |  | _ | _ | _ | _ | _ | _ |
| Syllidae | 130.7 (± 12.1) | 148.3 (± 56.2) | 160.0 (± 18.2) | 80.7 (± 24.3) | 104.0 (± 50.8) | 24.3 (± 5.6) |  | 0.3 (± 0.3) | 4.7 (± 2.7) | 1.7 (± 0.9) | 1.3 (± 0.3) | _ | 12.0 (± 4.9) |
| Nereididae | 21.7 (± 8.7) | 33.0 (± 5.5) | 16.7 (± 5.9) | 11.3 (± 7.4) | 18.3 (± 4.1) | 0.3 (± 0.3) |  | _ | 1.0 (± 1.0) | _ | 0.3 (± 0.3) | 0.3 (± 0.3) | 3.7 (± 0.9) |
| Oweniidae | _ | 0.3 (± 0.3) | _ | _ | 0.3 (± 0.3) | 1.0 (± 0.6) |  | _ | _ | _ | 0.3 (± 0.3) | _ | 2.3 (± 1.9) |
| Cirratulidae | _ | 1.3 (± 0.3) | 1.0 (± 0.6) | 1.3 (± 0.9) | 2.3 (± 0.9) | 0.3 (± 0.3) |  | 0.3 (± 0.3) | _ | _ | _ | _ | 2.3 (± 1.5) |
| Orbinidae | _ | 0.7 (± 0.3) | _ | _ | 2.3 (± 1.2) | _ |  | 0.3 (± 0.3) | _ | 0.3 (± 0.3) | _ | _ | 0.7 (± 0.7) |
| Flabelligeridae | 0.3 (± 0.3) | 0.7 (± 0.3) | 0.3 (± 0.3) | _ | 1.3 (± 1.3) | _ |  | _ | 0.3 (± 0.3) | _ | _ | _ | _ |
| Nephytidae | _ | 0.7 (± 0.3) | _ | 0.3 (± 0.3) | 0.3 (± 0.3) | _ |  | _ | _ | _ | _ | _ | _ |
| Mangelonidae | 0.3 (± 0.3) | 0.3 (± 0.3) | _ | _ | 0.7 (± 0.7) | _ |  | _ | _ | _ | _ | _ | _ |
| Capitellidae | 1.7 (± 1.7) | 2.3 (± 0.3) | 1.7 (± 0.7) | 0.3 (± 0.3) | 1.7 (± 0.9) | 0.7 (± 0.3) |  | _ | _ | _ | 0.3 (± 0.3) | 0.3 (± 0.3) | 3.0 (± 2.5) |
| Paraonidae | 1.7 (± 1.2) | 6.0 (± 0.6) | 4.0 (± 1.2) | 0.7 (± 0.7) | 9.3 (± 3.8) | 3.7 (± 0.7) |  | _ | _ | _ | _ | _ | _ |
| Onuphidae | 0.3 (± 0.3) | 3.0 (± 2.1) | 4.7 (± 2.6) | 13.0 (± 2.1) | 0.7 (± 0.7) | _ |  | _ | _ | _ | _ | _ | _ |
| Iospilidae | 0.3 (± 0.3) | _ | _ | _ | _ | 0.3 (± 0.3) |  | _ | _ | 0.3 (± 0.3) | _ | _ | _ |
| Dorvilleidae | 4.7 (± 1.9) | 3.0 (± 1.2) | 1.3 (± 0.3) | _ | 2.0 (± 0.6) | 0.7 (± 0.3) |  | _ | _ | 0.3 (± 0.3) | _ | _ | 0.3 (± 0.3) |
| Ampharetidae | 0.3 (± 0.3) | _ | 0.3 (± 0.3) | _ | 0.3 (± 0.3) | _ |  | _ | _ | _ | _ | _ | _ |
| Sternaspidae | 0.7 (± 0.3) | 0.7 (± 0.7) | _ | _ | 0.3 (± 0.3) | _ |  | _ | _ | _ | _ | _ | _ |
| Aphroditidae | 0.7 (± 0.3) | 0.7 (± 0.3) | 0.3 (± 0.3) | 1.3 (± 0.7) | 0.7 (± 0.3) | _ |  | _ | _ | _ | _ | _ | _ |
| Hesionidae | 2.3 (± 0.7) | 1.3 (± 1.3) | 0.3 (± 0.3) | 0.3 (± 0.3) | 1.0 (± 1.0) | _ |  | _ | _ | _ | _ | _ | _ |
| *Maldane* sp | 0.7 (± 0.3) | 0.7 (± 0.3) | 0.7 (± 0.7) | 0.7 (± 0.7) | _ | _ |  | _ | _ | _ | _ | _ | _ |
| *Lysidice* sp | 18.3 (± 3.7) | 19.0 (± 3.6) | 15.3 (± 1.2) | 28.0 (± 3.5) | 13.3 (± 5.6) | 1.3 (± 0.9) |  | _ | _ | _ | _ | _ | _ |
| *Eunice* sp | 3.3 (± 1.8) | 3.7 (± 0.7) | 5.0 (± 2.6) | 4.0 (± 1.7) | 4.0 (± 0.6) | 0.7 (± 0.3) |  | _ | 0.3 (± 0.3) | _ | _ | _ | 0.7 (± 0.3) |
| *Palolo* sp | 0.7 (± 0.3) | 0.3 (± 0.3) | 0.3 (± 0.3) | 2.0 (± 0.6) | 5.3 (± 3.8) | 0.3 (± 0.3) |  | _ | _ | _ | _ | _ | _ |
| *Marphysa* sp | 0.3 (± 0.3) | 0.7 (± 0.7) | 0.7 (± 0.7) | _ | _ | _ |  | _ | _ | _ | _ | _ | _ |
| *Megalomma* sp | 13.3 (± 3.3) | 7.0 (± 1.7) | 26.3 (± 10.0) | 12.0 (± 7.0) | 8.3 (± 3.8) | 0.7 (± 0.3) |  | _ | _ | _ | _ | _ | _ |
| *Hydroides* sp | 0.7 (± 0.7) | 1.3 (± 0.7) | 1.0 (± 1.0) | 1.0 (± 0.6) | _ | _ |  | _ | _ | _ | _ | _ | _ |
| *Pseudovermilia* sp | 21.3 (± 5.4) | 12.3 (± 1.2) | 13.0 (± 7.8) | 5.7 (± 1.8) | 3.0 (± 1.0) | 1.7 (± 0.7) |  | _ | _ | 0.3 (± 0.3) | _ | _ | 1.0 (± 0.6) |
| *Vermiliopsis* sp | 3.0 (± 1.5) | 3.0 (± 0.6) | 3.0 (± 1.7) | 0.7 (± 0.3) | 0.3 (± 0.3) | 0.3 (± 0.3) |  | _ | _ | _ | _ | _ | _ |
| *Anaitides* sp | 5,3 (± 0.9) | 4.0 (± 1.2) | 6.0 (± 1.2) | 3.3 (± 2.8) | 1.7 (± 0.3) | _ |  | _ | _ | _ | _ | _ | _ |
| *Phyllodoce* sp1 | 9.3 (± 3.7) | 4.3 (± 2.3) | 5.3 (± 1.7) | 1.7 (± 0.9) | 3.3 (± 0.3) | _ |  | _ | _ | _ | _ | _ | 1.7 (± 0.7) |
| *Phyllodoce* sp2 | 1.7 (± 0.7) | 1.7 (± 0.9) | _ | 0.7 (± 0.7) | 1.0 (± 0.6) | _ |  | _ | _ | _ | _ | _ | _ |
| *Pectinaria* sp | _ | _ | 0.3 (± 0.3) | _ | 0.3 (± 0.3) | 0.3 (± 0.3) |  | _ | _ | _ | _ | _ | _ |
| *Arabella* sp | 1.3 (± 0.7) | 3.7 (± 2.7) | _ | _ | 1.0 (± 0.6) | 1.3 (± 0.7) |  | _ | _ | _ | _ | _ | 0.3 (± 0.3) |
| *Oenone* sp | 0.3 (± 0.3) | 0.7 (± 0.7) | _ | _ | 0.7 (± 0.7) | _ |  | _ | _ | _ | _ | _ | _ |
| *Lumbrineris* sp | 2.0 (± 1.5) | 4.0 (± 0.6) | 4.3 (±1.7) | 2.0 (± 1.5) | 1.0 (± 0.6) | _ |  | _ | _ | 0.3 (± 0.3) | _ | _ | 0.3 (± 0.3) |
| *Notopygos* sp | 5.3 (± 1.9) | 16.0 (± 5.8) | 3.0 (± 1.2) | 2.0 (± 0.6) | 1.3 (± 1.3) | 0.3 (± 0.3) |  | _ | 0.3 (± 0.3) | _ | _ | _ | 0.3 (± 0.3) |
| *Glycinde multidens* | _ | _ | 0.3 (± 0.3) | _ | _ | _ |  | _ | _ | _ | _ | _ | _ |
| *Glycera lapidum* | 0.3 (± 0.3) | 2.7 (± 2.2) | _ | _ | _ | _ |  | _ | _ | _ | _ | _ | _ |
| *Goniadides* sp | 2.0 (± 1.5) | 2.0 (± 0.6) | 1.7 (± 1.2) | 0.6 (± 0.3) | 1.0 (± 0.6) | _ |  | _ | _ | _ | _ | _ | 4.7 (± 2.6) |
| *Hemipodia californiensis* | _ | 0.3 (± 0.3) | _ | 0.7 (± 0.7) | _ | _ |  | _ | _ | _ | _ | _ | _ |
| *Glycera* sp | _ | 2.7 (± 0.9) | 0.7 (± 0.7) | _ | 0.3 (± 0.3) | _ |  | _ | 0.3 (± 0.3) | 0.3 (± 0.3) | 0.7 (± 0.3) | 0.3 (± 0.3) | 0.3 (± 0.3) |
| *Hemipodia* sp | 0.3 (± 0.3) | 0.7 (± 0.7) | _ | _ | _ | _ |  | _ | _ | _ | _ | _ | _ |
| *Harmothoe* sp | 7.7 (± 2.9) | 14.0 (± 0.6) | 11.0 (± 3.6) | 11.0 (± 4.6) | 16.3 (± 3.8) | _ |  | _ | _ | 0.3 (± 0.3) | _ | 0.3 (± 0.3) | 0.7 (± 0.7) |
| *Lepidonotus* sp | 2.0 (± 0.6) | 3.0 (± 1.5) | 2.0 (± 1.5) | 1.0 (± 1.0) | 1.7 (± 0.3) | _ |  | _ | _ | _ | _ | _ | _ |
| *Polynoidae* sp4 | 10.7 (± 7.2) | 26.0 (± 4.2) | 8.3 (± 2.6) | 1.0 (± 0.6) | 1.0 (± 1.0) | _ |  | _ | 1.3 (± 0.9) | _ | _ | _ | 1.3 (± 0.7) |
| *Polynoidae* sp5 | 24.0 (± 2.3) | 11.3 (± 2.7) | 15.3 (± 4.7) | 7.3 (± 5.8) | 9.7 (± 4.8) | 0.3 (± 0.3) |  | _ | _ | _ | 0.3 (± 0.3) | 0.3 (± 0.3) | 2.3 (± 1.2) |
| *Polynoidae* sp7 | 0.3 (± 0.3) | _ | _ | 0.7 (± 0.7) | 1.7 (± 0.9) | _ |  | _ | _ | _ | _ | _ | _ |
| *Minuspio* sp | 0.7 (± 0.3) | 1.0 (± 0.0) | _ | _ | 2.0 (± 1.5) | 0.3 (± 0.3) |  | _ | _ | _ | _ | _ | 4.0 (± 3.1) |
| *Terebellides* sp | 4.3 (± 1.8) | 1.0 (± 0.6) | 2.3 (± 0.3) | 0.7 (± 0.7) | 3.7 (± 1.3) | _ |  | _ | _ | _ | _ | _ | _ |
| *Pholoe* sp | 1.3 (± 0.9) | 2.3 (± 1.9) | 1.0 (± 0.0) | 2.7 (± 1.7) | 2.3 (± 0.3) | _ |  | _ | _ | _ | _ | _ | _ |
| *Polydora* sp | 1.0 (± 0.6) | 0.7 (± 0.7) | 0.3 (± 0.3) | 2.7 (± 2.7) | 0.3 (± 0.3) | _ |  | _ | _ | _ | _ | _ | _ |
| *Prionospio* sp | 1.3 (± 1.3) | _ | 0.3 (± 0.3) | _ | 1.3 (± 0.9) | _ |  | _ | _ | _ | _ | _ | _ |
| *Longosomatidae* sp | _ | _ | 0.7 (± 0.7) | _ | _ | _ |  | _ | _ | _ | _ | _ | _ |
| *Eurythoe* sp | _ | 0.3 (± 0.3) | _ | _ | _ | _ |  | _ | _ | _ | _ | _ | _ |
| Oligochaeta | 1.3 (± 1.3) | _ | 0.7 (± 0.7) | _ | 2.0 (± 2.0) | _ |  | _ | _ | _ | _ | _ | _ |
| **Crustacea**  Mithracidae | 1.0 (± 0.6) | _ | 0.3 (± 0.3) | 0.3 (± 0.3) | 1.3 (± 0.3) | _ |  | _ | _ | _ | _ | _ | _ |
| Xanthidae sp1 | _ | 0.3 (± 0.3) | 1.0 (± 0.0) | 0.3 (± 0.3) | 1.0 (± 0.0) | _ |  | _ | _ | _ | _ | _ | _ |
| Teleophrys sp | 0.7 (± 0.3) | _ | 0.7 (± 0.7) | 0.3 (± 0.3) | 2.3 (± 1.2) | _ |  | _ | _ | _ | _ | _ | _ |
| Brachiura n id | 0.3 (± 0.3) | _ | _ | _ | 0.7 (± 0.3) | _ |  | _ | _ | _ | _ | _ | 0.3 (± 0.3) |
| Pilumnidae | 1.3 (±0.9) | 0.3 (± 0.3) | 0.7 (± 0.3) | 0.3 (± 0.3) | 2.0 (± 1.0) | _ |  | _ | _ | _ | _ | _ | _ |
| *Stenorhynchus* sp | _ | 0.3 (± 0.3) | _ | _ | 1.0 (± 1.0) | _ |  | _ | _ | _ | _ | _ | _ |
| Xanthidae sp2 | 0.3 (± 0.3) | 0.3 (± 0.3) | 0.3 (± 0.3) | 0.7 (± 0.3) | 1.3 (± 0.3) | _ |  | _ | _ | _ | _ | _ | _ |
| Ocypodidae | _ | _ | _ | _ | _ | _ |  | _ | 0.3 (± 0.3) | _ | _ | _ | _ |
| *Epialtus* sp | _ | _ | _ | _ | 0.3 (± 0.3) | _ |  | _ | _ | _ | _ | 0.3 (± 0.3) | 0.3 (± 0.3) |
| *Ebalia* sp | _ | _ | _ | 0.3 (± 0.3) | _ | _ |  | _ | _ | _ | _ | _ | _ |
| Porcellanidae | _ | _ | 0.7 (± 0.3) | _ | _ | _ |  | _ | _ | _ | _ | _ | _ |
| *Paractaea* sp | _ | _ | _ | 0.3 (± 0.3) | _ | _ |  | _ | _ | _ | _ | _ | _ |
| *Paguropsina* sp | _ | _ | 1.3 (± 1.3) | _ | 0.7 (± 0.7) | _ |  | _ | _ | _ | _ | _ | _ |
| *Coenobitidae* sp | _ | _ | 0.3 (± 0.3) | _ | _ | _ |  | _ | _ | _ | _ | 0.3 (± 0.3) | _ |
| *Aniculus* sp | 0.3 (± 0.3) | 3.0 (± 1.2) | 3.7 (± 1.5) | 2.3 (± 0.9) | 9.7 (± 4.3) | _ |  | _ | _ | _ | 0.3 (± 0.3) | _ | _ |
| Caridea | 2.7 (± 0.9) | 5.0 (± 4.0) | 7.0 (± 2.6) | 3.7 (± 0.3) | 14.7 (± 1.7) | _ |  | _ | 0.3 (± 0.3) | _ | _ | _ | 4.7 (± 3.7) |
| Mysidae sp1 | 2.0 (± 1.0) | _ | 1.3 (± 0.7) | 2.0 (± 2.0) | 3.3 (± 3.3) | _ |  | _ | _ | _ | _ | _ | _ |
| Mysidae sp2 | 2.0 (± 1.0) | 0.3 (± 0.3) | 1.7 (± 0.7) | 0.3 (± 0.3) | 2.0 (± 1.0) | _ |  | _ | _ | _ | _ | _ | _ |
| Decapoda n id | 0.3 (± 0.3) | _ | _ | _ | _ | _ |  | _ | _ | _ | _ | _ | _ |
| Aristiidae | 4.0 (± 2.1) | 4.7 (± 2.3) | 3.0 (± 1.7) | 1.3 (± 0.9) | 8.3 (± 2.7) | _ |  | _ | _ | _ | _ | 0.3 (± 0.3) | 1.7 (± 1.2) |
| Caprellidae | 0.7 (± 0.7) | 0.3 (± 0.3) | 2.3 (± 1.2) | 0.7 (± 0.3) | 3.7 (± 2.3) | _ |  | _ | _ | _ | _ | 0.7 (± 0.7) | _ |
| *Dexamine spinosa* | 2.0 (± 1.5) | 0.7 (± 0.7) | 1.0 (± 0.6) | _ | 0.3 (± 0.3) | _ |  | _ | _ | _ | _ | _ | _ |
| Gammaridae | 65.7 (± 7.9) | 102.6 (± 54.9) | 141.3 (± 19.7) | 452.7 (± 157.7) | 310.3 (± 123.7) | 111.3 (± 31.6) |  | _ | 1.7 (± 1.7) | 2.3 (± 1.9) | 0.3 (± 0.3) | 1.0 (± 1.0) | 3.0 (± 2.1) |
| Melitidae | 7.0 (± 3.2) | 8.7 (± 4.7) | 9.7 (± 1.9) | 24.7 (± 4.8) | 22.7 (± 11.3) | 12.7 (± 10.7) |  | _ | 1.7 (± 0.9) | _ | _ | 11.3 (± 11.3) | _ |
| Amphilochidae | 8.3 (± 1.8) | 5.7 (± 1.9) | 3.0 (± 3.0) | 4.0 (± 3.5) | 5.7 (± 4.7) | 0.3 (± 0.3) |  | _ | _ | _ | _ | _ | _ |
| *Elasmopus* sp | 1.7 (± 1.2) | 6.0 (± 1.2) | 11.3 (± 3.7) | 21.3 (± 9.5) | 4.3 (± 2.8) | 4.0 (± 2.6) |  | 0.7 (± 0.7) | 1.0 (± 1.0) | 0.3 (± 0.3) | 0.3 (± 0.3) | _ | 1.7 (± 1.2) |
| *Leptochelia* sp | 16.7 (± 1.5) | 10.0 (± 2.9) | 15.3 (± 7.0) | 11.0 (± 3.8) | 8.0 (± 3.2) | 0.3 (± 0.3) |  | _ | 0.3 (± 0.3) | 1.3 (± 1.3) | 1.7 (± 1.2) | 0.7 (± 0.7) | 4.7 (± 0.7) |
| Tanaidæ | 8.0 (± 3.2) | 10.7 (± 2.0) | 11.7 (± 5.0) | 9.0 (± 7.5) | 3.7 (± 1.2) | 0.3 (± 0.3) |  | 0.3 (± 0.3) | 3.3 (± 3.3) | 0.3 (± 0.3) | 0.3 (± 0.3) | _ | 4.3 (± 1.2) |
| Anthuridae | 9.7 (± 4.3) | 19.3 (± 4.7) | 12.7 (± 1.9) | 9.0 (± 2.1) | _ | 2.0 (± 0.6) |  | _ | _ | _ | _ | 0.3 (± 0.3) | 3.3 (± 2.8) |
| Janiridae | 11.7 (± 6.2) | 17.0 (±4.0) | 11.3 (± 2.6) | 6.3 (± 1.2) | 11.7 (± 6.2) | 0.3 (± 0.3) |  | _ | 0.7 (± 0.7) | 0.3 (± 0.3) | 0.7 (± 0.7) | _ | 2.3 (± 0.7) |
| Cirolanidae | 2.3 (± 1.3) | 3.7 (± 2.2) | 1.0 (± 0.6) | _ | 3.0 (± 1.0) | 0.3 (± 0.3) |  | _ | 0.3 (± 0.3) | _ | 0.3 (± 0.3) | _ | 1.0 (± 0.6) |
| Gnathiidae sp1 | 3.3 (± 0.3) | 0.7 (± 0.3) | 0.7 (± 0.3) | _ | 2.3 (± 1.3) | _ |  | 0.3 (± 0.3) | _ | _ | _ | 0.3 (± 0.3) | 0.3 (± 0.3) |
| Gnathiidae sp2 | 1.3 (± 0.3) | _ | _ | _ | _ | _ |  | _ | _ | _ | _ | _ | _ |
| Apseudidae | 15.7 (± 4.7) | 9.0 (± 4.9) | 4.7 (± 3.7) | 0.7 (± 0.7) | 5.0 (± 2.5) | _ |  | _ | _ | _ | _ | _ | _ |
| *Oniscus* sp | _ | _ | 0.3 (± 0.3) | _ | _ | _ |  | _ | _ | _ | _ | _ | _ |
| Ostracoda | 11.7 (± 4.1) | 2.7 (± 0.7) | 1.7 (± 0.7) | 0.3 (± 0.3) | _ | _ |  | 13.3 (± 4.9) | 12.0 (± 0.6) | 13.0 (± 2.5) | 6.3 (± 3.8) | 1.7 (± 1.2) | 26.3 (± 2.8) |
| Cumacea | 2.3 (± 0.9) | 3.0 (± 0.6) | 2.0 (± 1.0) | 3.7 (± 2.7) | 5.0 (± 2.5) | 0.7 (± 0.3) |  | 0.3 (± 0.3) | 1.7 (± 0.9) | _ | 0.3 (± 0.3) | 0.7 (± 0.3) | 2.3 (± 0.9) |
| Valvifera | _ | 0.3 (0.3) | _ | _ | 1.7 (± 0.7) | 0.7 (± 0.3) |  | _ | _ | _ | 0.3 (± 0.3) | _ | 1.0 (± 1.0) |
| *Isochnochiton* sp | 7.0 (± 1.0) | 14.3 (± 2.6) | 15.7 (± 4.4) | 2.7 (± 2.2) | 2.3 (± 0.9) | 0.3 (± 0.3) |  | _ | _ | 0.3 (± 0.3) | _ | _ | 0.3 (± 0.3) |
| **Mollusca - Gastropoda**  Nudibranchia | 1.3 (± 0.9) | _ | 1.0 (± 1.0) | 0.3 (± 0.3) | 1.0 (± 0.6) | _ |  | _ | _ | _ | _ | _ | _ |
| Mollusca n id | 9.0 (± 6.0) | 13.0 (± 2.3) | 9.0 (± 8.0) | 5.3 (± 0.3) | 1.0 (± 0.6) | 2.7 (± 2.7) |  | _ | _ | _ | _ | _ | _ |
| Rissoidae | _ | _ | _ | 0.3 (± 0.3) | _ | _ |  | 1.3 (± 0.9) | 1.3 (± 0.9) | _ | _ | _ | _ |
| Pyramidellidae | _ | 0.3 (± 0.3) | _ | _ | _ | _ |  | _ | _ | _ | _ | _ | _ |
| Turridae | _ | _ | _ | _ | 1.0 (± 0.6) | _ |  | _ | _ | _ | _ | _ | _ |
| Hydrobiidae | _ | 0,7 (± 0.3) | _ | _ | 0.3 (± 0.3) | _ |  | _ | 0.3 (± 0.3) | 0.3 (± 0.3) | _ | 0.3 (± 0.3) | _ |
| Marginellidae | _ | _ | _ | _ | _ | _ |  | _ | _ | 0.3 (± 0.3) | _ | _ | _ |
| Lepetidae | _ | _ | _ | 0.3 (± 0.3) | _ | _ |  | _ | _ | _ | _ | _ | _ |
| Lottiidae | _ | _ | 0.3 (± 0.3) | _ | 0.3 (± 0.3) | _ |  | _ | _ | _ | _ | _ | _ |
| Phasianellidae | _ | _ | _ | _ | 0.3 (± 0.3) | _ |  | _ | _ | _ | _ | _ | _ |
| Eulimidae | 0.3 (± 0.3) | _ | _ | 0.3 (± 0.3) | 0.3 (± 0.3) | _ |  | 0.3 (± 0.3) | _ | _ | 0.3 (± 0.3) | 0.7 (± 0.3) | _ |
| Costellariidae | 0.3 (± 0.3) | 0.3 (± 0.3) | 0.3 (± 0.3) | _ | 0.7 (± 0.7) | _ |  | _ | _ | _ | _ | _ | _ |
| Architectonicidae | _ | _ | _ | _ | 0.3 (± 0.3) | _ |  | _ | _ | _ | _ | _ | _ |
| Trochidae | 0.3 (± 0.3) | 0.3 (± 0.3) | 0.3 (± 0.3) | _ | 0.3 (± 0.3) | _ |  | _ | _ | _ | _ | _ | _ |
| *Meioceras* sp | 1.0 (± 0.0) | 1.0 (± 1.0) | _ | _ | 1.0 (± 1.0) | _ |  | 2.3 (± 0.9) | 1.7 (± 0.9) | 0.3 (± 0.3) | 1.0 (± 1.0) | 0.3 (± 0.3) | 1.7 (± 1.7) |
| Gastropoda n id | _ | _ | 0.7 (± 0.3) | _ | _ | _ |  | _ | _ | 0.3 (± 0.3) | 0.3 (± 0.3) | 0.3 (± 0.3) | _ |
| Capulidae | 1.7 (± 0.3) | 0.7 (± 0.7) | 0.7 (± 0.3) | 0.3 (± 0.3) | 0.3 (± 0.3) | _ |  | _ | 0.3 (± 0.3) | _ | _ | _ | _ |
| Coralliophilidae | 0.3 (± 0.3) | _ | _ | _ | _ | _ |  | _ | _ | _ | _ | _ | _ |
| Naticidae | 1.7 (± 1.7) | 0.3 (± 0.3) | 0.3 (± 0.3) | _ | _ | _ |  | _ | _ | _ | _ | 0.3 (± 0.3) | 0.3 (± 0.3) |
| Nassariidae | _ | _ | _ | _ | _ | _ |  | _ | _ | 1.0 (± 1.0) | 1.0 (± 1.0) | _ | _ |
| Planaxidae | 1.0 (± 1.0) | 0.7 (± 0.3) | 0.3 (± 0.3) | _ | 0.7 (± 0.7) | _ |  | 0.3 (± 0.3) | _ | 0.3 (± 0.3) | 1.0 (± 1.0) | _ | _ |
| Cerithiopsidae | 2.3 (± 1.9) | 1.0 (± 1.0) | 0.3 (± 0.3) | 0.3 (± 0.3) | 1.3 (± 1.3) | _ |  | 0.3 (± 0.3) | _ | 0.3 (± 0.3) | _ | 0.7 (± 0.3) | _ |
| Gastropoda | _ | _ | 0.3 (± 0.3) | _ | _ | _ |  | _ | _ | _ | _ | _ | _ |
| **Mollusca - Bivalvia**  Pectinidae | 1.3 (± 0.7) | 2.0 (± 0.6) | _ | _ | 2.0 (± 0.6) | _ |  | _ | _ | _ | _ | _ | _ |
| Veneridae | 9.7 (± 1.8) | 5.7 (± 2.4) | 2.7 (± 1.8) | 0.7 (± 0.7) | 2.0 (± 1.0) | 0.3 (± 0.3) |  | 1.0 (± 1.0) | 1.0 (± 0.6) | 0.7 (± 0.7) | 0.3 (± 0.3) | 0.7 (± 0.7) | 1.0 (± 0.0) |
| Crassatellidae | 1.7 (± 1.7) | 1.3 (± 0.9) | 1.0 (± 0.6) | 0.3 (± 0.3) | 0.3 (± 0.3) | _ |  | 0.7 (± 0.3) | 0.7 (± 0.7) | _ | _ | 0.7 (± 0.3) | 0.3 (± 0.3) |
| Tellinidae | 3.3 (± 1.7) | 6.3 (± 0.9) | 7.3 (± 0.7) | 2.7 (± 2.2) | 2.7 (± 0.9) | 1.7 (± 0.3) |  | _ | 0.3 (± 0.3) | _ | _ | _ | _ |
| Mesodesma | 1.0 (± 0.6) | 1.7 (± 0.3) | 0.3 (± 0.3) | 0.3 (± 0.3) | 1.0 (± 0.6) | _ |  | _ | _ | _ | _ | _ | _ |
| Cardiidae | 5.7 (± 4.2) | 2.7 (± 1.2) | 0.3 (± 0.3) | 0.3 (± 0.3) | 3.3 (± 1.2) | 0.3 (± 0.3) |  | 2.7 (± 1.3) | 0.3 (± 0.3) | 1.3 (± 0.7) | _ | _ | 1.3 (± 1.3) |
| Mytilidae | _ | 0.7 (± 0.3) | 0.3 (± 0.3) | _ | 0.3 (± 0.3) | 0.3 (± 0.3) |  | _ | _ | _ | _ | _ | _ |
| Bivalva n id | 0.7 (± 0.3) | 1.3 (± 1.3) | 0.3 (± 0.3) | _ | _ | _ |  | _ | _ | _ | _ | _ | _ |
| Isognomon | _ | 0.7 (± 0.3) | _ | 1.7 (± 0.9) | _ | _ |  | _ | _ | _ | _ | _ | _ |
| Thraciidae | _ | 0.7 (± 0.3) | _ | _ | _ | 0.3 (± 0.3) |  | _ | _ | _ | _ | _ | _ |
| Lucinidae | _ | 0.3 (± 0.3) | 0.7 (± 0.7) | 0.3 (± 0.3) | 1.0 (± 0.6) | 0.7 (± 0.7) |  | _ | _ | _ | _ | 0.3 (± 0.3) | 1.3 (± 1.3) |
| Chamidae | _ | _ | 0.3 (± 0.3) | 0.3 (± 0.3) | 0.3 (± 0.3) | _ |  | _ | _ | _ | _ | _ | _ |
| Arcidae | 0.3 (± 0.3) | _ | 1.0 (± 0.0) | 1.3 (± 0.9) | 1.0 (± 0.0) | _ |  | 0.3 (± 0.3) | _ | _ | _ | _ | _ |
| **Echinodermata**  Holothuroidea | 1.3 (± 1.3) | 0.3 (± 0.3) | 0.3 (± 0.3) | _ | 1.0 (± 0.6) | 0.7 (± 0.3) |  | _ | _ | _ | _ | 0.3 (± 0.3) | 1.0 (± 0.6) |
| Psolidae | 7.7 (± 3.8) | 7.0 (± 4.0) | 3.0 (± 1.2) | _ | 1.3 (± 0.9) | 0.3 (± 0.3) |  | _ | _ | _ | _ | _ | _ |
| Dendrochirotida | _ | 0.7 (± 0.7) | 0.3 (± 0.3) | 0.3 (± 0.3) | 1.0 (± 0.0) | _ |  | _ | _ | _ | _ | _ | _ |
| Amphiuridae | 3.0 (± 0.6) | 14.0 (± 5.0) | 12.0 (± 4.6) | 22.0 (± 6.0) | 15.3 (± 0.9) | 5.7 (± 1.7) |  | _ | _ | 0.7 (± 0.7) | _ | 4.0 (± 0.0) | 2.7 (± 2.7) |
| *Amphipolis* sp | 0.3 (± 0.3) | 1.0 (± 1.0) | _ | 1.7 (± 1.7) | 0.7 (± 0.7) | _ |  | _ | _ | _ | _ | _ | _ |
| *Ophioderma* sp | _ | 0.3 (± 0.3) | _ | _ | _ | _ |  | _ | _ | _ | _ | _ | _ |
| *Ophiothrix* sp | 1.0 (± 0.6) | 3.0 (± 1.5) | 4.7 (± 2.2) | 1.7 (± 0.9) | 3.3 (± 2.4) | _ |  | 0.3 (± 0.3) | _ | _ | _ | _ | _ |
| Ophionereididae | 0.7 (± 0.7) | 1.0 (± 0.6) | 0.3 (± 0.3) | 1.0 (± 0.6) | 0.7 (± 0.7) | _ |  | _ | _ | _ | _ | _ | _ |
| Asteroidae | _ | 0.3 (± 0.3) | _ | _ | 0.3 (± 0.3) | _ |  | _ | _ | _ | _ | _ | _ |
| Crinoidae | _ | _ | _ | _ | 1.0 (± 0.0) | _ |  | _ | _ | _ | _ | _ | _ |
| Echinoidea sp1 | 0.3 (± 0.3) | 0.7 (± 0.7) | 0.3 (± 0.3) | 0.7 (± 0.3) | _ | _ |  | _ | _ | _ | _ | _ | _ |
| Echinoidea sp2 | _ | _ | _ | _ | _ | _ |  | _ | _ | 0.3 (± 0.3) | _ | _ | _ |
| **Other**  Nemertea | 1.0 (± 0.0) | 1.7 (± 0.3) | 1.0 (± 1.0) | _ | 2.3 (± 0.7) | 0.3 (± 0.3) |  | _ | _ | _ | _ | _ | _ |
| Sipuncula | 27.3 (± 8.1) | 58.3 (± 8.2) | 38.3 (± 4.1) | 13.3 (± 3.8) | 5.7 (± 1.8) | 1.7 (± 1.2) |  | _ | _ | _ | _ | _ | 2.7 (± 0.3) |
| Echiura | 0.3 (± 0.3) | _ | 1.7 (± 1.7) | _ | 1.3 (± 0.7) | _ |  | _ | _ | _ | _ | _ | _ |
| Nematoda | 2.7 (± 1.5) | _ | 2.7 (± 2.7) | _ | 5.0 (± 5.0) | _ |  | _ | _ | _ | _ | _ | _ |
| Platyhelminthes | _ | _ | _ | _ | 0.3 (± 0.3) | _ |  | _ | _ | _ | _ | _ | _ |


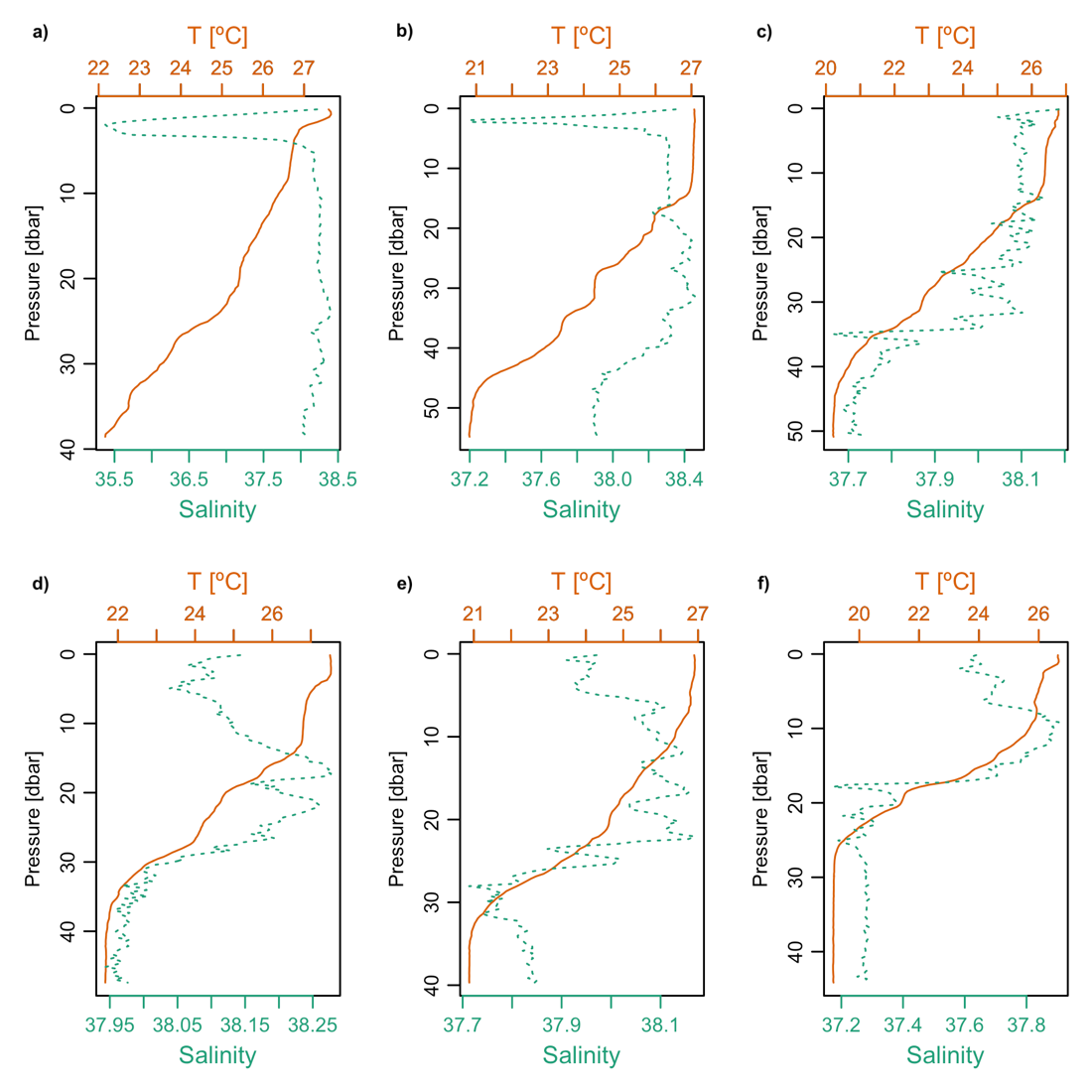


**Figure S1.** Vertical profiles of temperature (ºC, orange solid line) and salinity (green dashed line) along a bathymetric gradient in the sampled stations during January 2019. a) H1, b) H2, c) H3, d) L1, e) L2 and f) L3


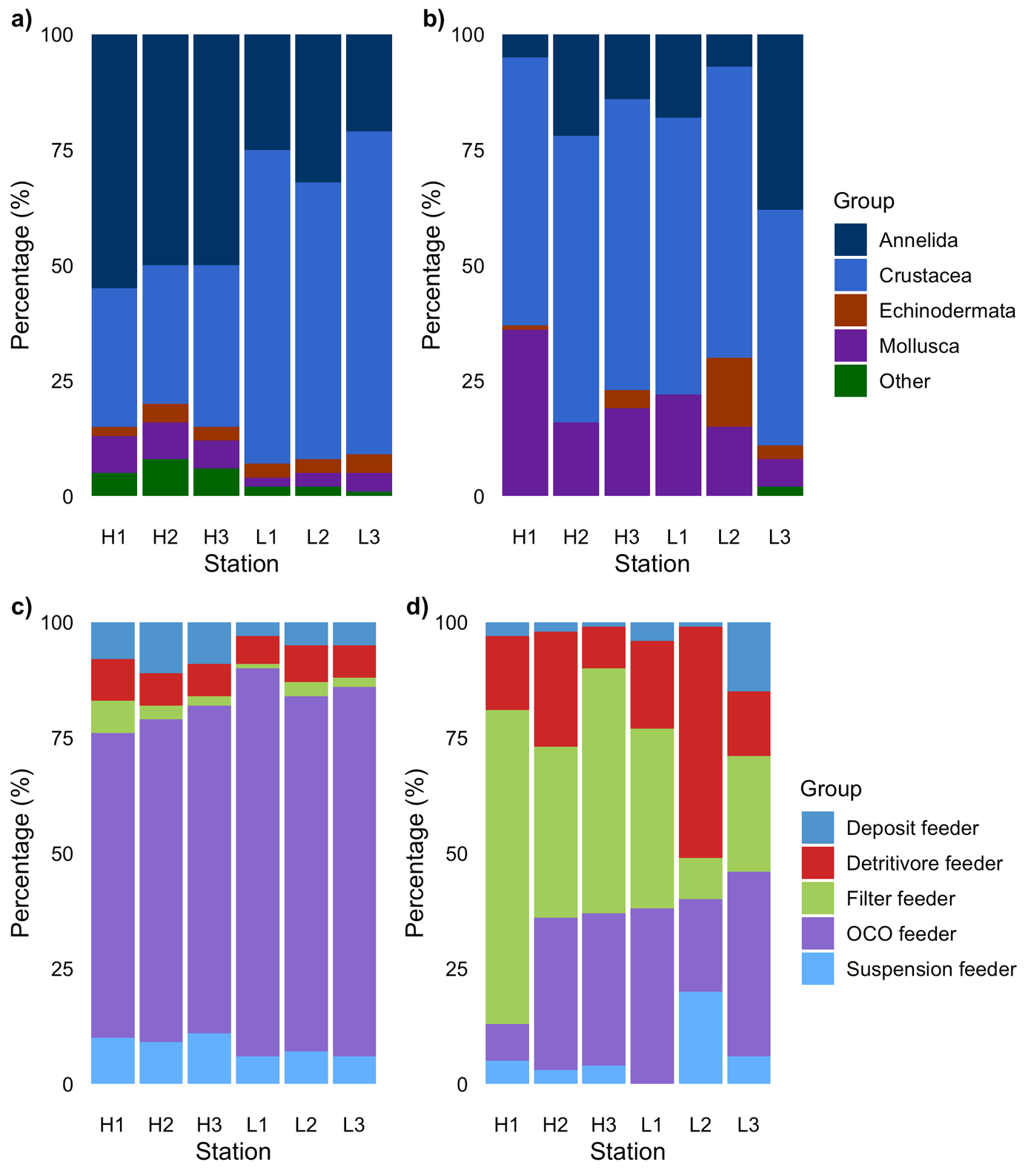


**Figure S2.** Relative abundance of macrofauna assemblages among RBs (a, c) and unconsolidated sediment stations (b, d). a) Taxonomic composition of macrofauna in rhodolith beds; b) Taxonomic composition of macrofauna in unconsolidated sediment under rhodoliths. c) relative abundance of macrofaunal trophic groups in RBs; d) relative abundance of macrofaunal trophic groups in unconsolidated sediment under rhodoliths.


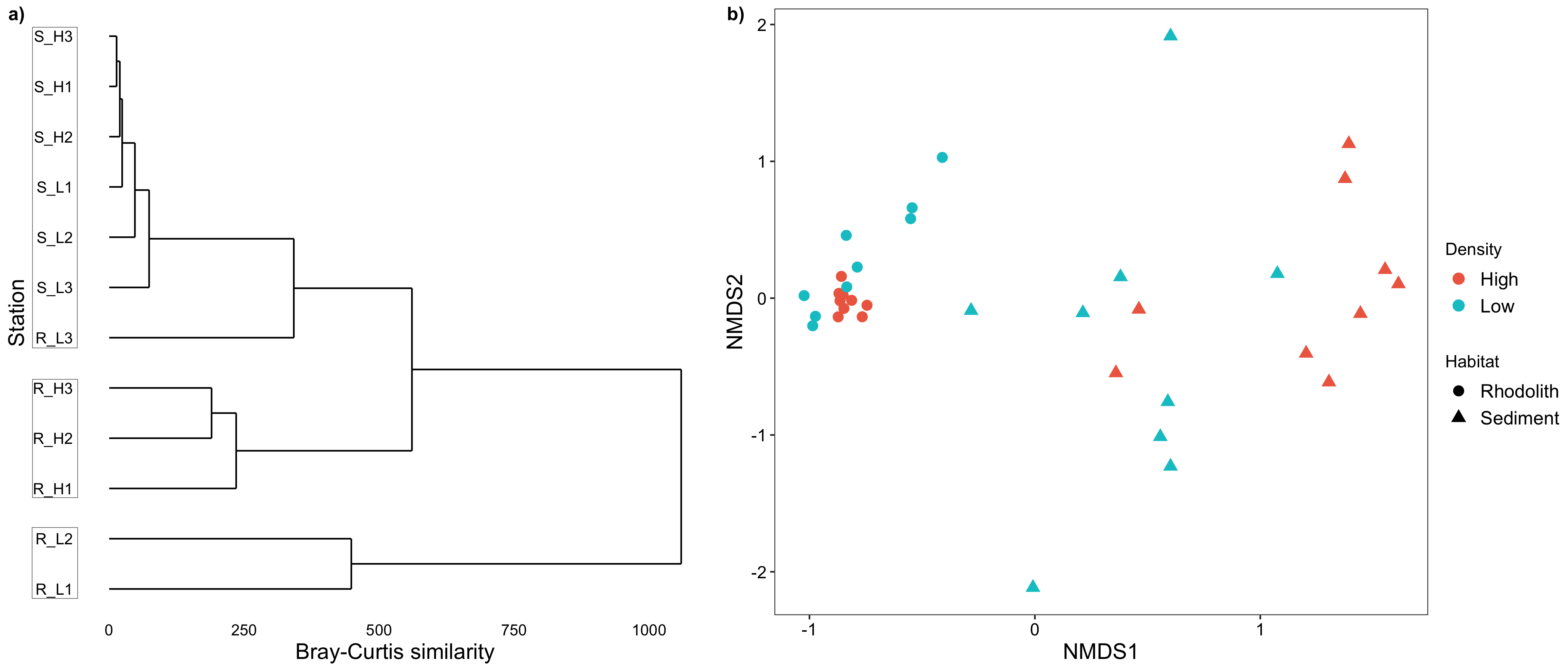


**Figure S3.** Bray-Curtis similarities indices of transformed benthic fauna abundances. a) Dendrogram using group average clustering across 6 stations between sampled stations through high and low-density rhodolith beds (R) and sediments underlying sampled beds (S). b) Plot of nMDS analysis for the similarities (Bray-Curtis) of the total abundance of the benthic macrofauna between the RBs and unconsolidated sediments below the beds. Red spheres represent high-density stations of RBs; Blue spheres represent low-density stations of RBs; Red triangles represent high-density stations in unconsolidated sediment; Blue triangles represent low-density stations in unconsolidated sediment. Test stress: 0.148.
